# A rare variant in *APOC3* is associated with plasma triglyceride and VLDL levels in Europeans

**DOI:** 10.1038/ncomms5871

**Published:** 2014-09-16

**Authors:** Nicholas J. Timpson, Klaudia Walter, Josine L. Min, Ioanna Tachmazidou, Giovanni Malerba, So-Youn Shin, Lu Chen, Marta Futema, Lorraine Southam, Valentina Iotchkova, Massimiliano Cocca, Jie Huang, Yasin Memari, Shane McCarthy, Petr Danecek, Dawn Muddyman, Massimo Mangino, Cristina Menni, John R. B. Perry, Susan M. Ring, Amadou Gaye, George Dedoussis, Aliki-Eleni Farmaki, Paul Burton, Philippa J. Talmud, Giovanni Gambaro, Tim D. Spector, George Davey Smith, Richard Durbin, J Brent Richards, Steve E. Humphries, Eleftheria Zeggini, Nicole Soranzo, UK1OK consortium members, Saeed Al Turki, Carl Anderson, Richard Anney, Dinu Antony, Maria Soler Artigas, Muhammad Ayub, Senduran Balasubramaniam, Jeffrey C. Barrett, Inês Barroso, Phil Beales, Jamie Bentham, Shoumo Bhattacharya, Ewan Birney, Douglas Blackwood, Martin Bobrow, Elena Bochukova, Patrick Bolton, Rebecca Bounds, Chris Boustred, Gerome Breen, Mattia Calissano, Keren Carss, Krishna Chatterjee, Lu Chen, Antonio Ciampi, Sebhattin Cirak, Peter Clapham, Gail Clement, Guy Coates, David Collier, Catherine Cosgrove, Tony Cox, Nick Craddock, Lucy Crooks, Sarah Curran, David Curtis, Allan Daly, Petr Danecek, George Davey Smith, Aaron Day-Williams, Ian N. M. Day, Thomas Down, Yuanping Du, Ian Dunham, Richard Durbin, Sarah Edkins, Peter Ellis, David Evans, Sadaf Faroogi, Ghazaleh Fatemifar, David R. Fitzpatrick, Paul Flicek, James Flyod, A Reghan Foley, Christopher S Franklin, Marta Futema, Louise Gallagher, Tom Gaunt, Matthias Geihs, Daniel Geschwind, Celia Greenwood, Heather Griffin, Detelina Grozeva, Xueqin Guo, Xiaosen Guo, Hugh Gurling, Deborah Hart, Audrey Hendricks, Peter Holmans, Bryan Howie, Jie Huang, Liren Huang, Tim Hubbard, Steve E. Humphries, Matthew E. Hurles, Pirro Hysi, David K. Jackson, Yalda Jamshidi, Tian Jing, Chris Joyce, Jane Kaye, Thomas Keane, Julia Keogh, John Kemp, Karen Kennedy, Anja Kolb-Kokocinski, Genevieve Lachance, Cordelia Langford, Daniel Lawson, Irene Lee, Monkol Lek, Jieqin Liang, Hong Lin, Rui Li, Yingrui Li, Ryan Liu, Jouko Lönnqvist, Margarida Lopes, Valentina Lotchkova, Daniel MacArthur, Jonathan Marchini, John Maslen, Mangino Massimo, Iain Mathieson, Gaëlle Marenne, Shane McCarthy, Peter McGuffin, Andrew McIntosh, Andrew G. McKechanie, Andrew McQuillin, Yasin Memari, Sarah Metrustry, Josine Min, Hannah Mitchison, Alireza Moayyeri, James Morris, Dawn Muddyman, Francesco Muntoni, Kate Northstone, Michael O'Donnovan, Alexandros Onoufriadis, Stephen O'Rahilly, Karim Oualkacha, Michael J. Owen, Aarno Palotie, Kalliope Panoutsopoulou, Victoria Parker, Jeremy R. Parr, Lavinia Paternoster, Tiina Paunio, Felicity Payne, John Perry, Olli Pietilainen, Vincent Plagnol, Lydia Quaye, Michael A. Quail, Lucy Raymond, Karola Rehnström, Brent Richards, Susan Ring, Graham R. S. Ritchie, Nicola Roberts, David B. Savage, Peter Scambler, Stephen Schiffels, Miriam Schmidts, Nadia Schoenmakers, Robert K. Semple, Eva Serra, Sally I. Sharp, Hasheem Shihab, So-Youn Shin, David Skuse, Kerrin Small, Nicole Soranzo, Lorraine Southam, Olivera Spasic-Boskovic, Tim Spector, David St Clair, Jim Stalker, Elizabeth Stevens, Beate St Pourcian, Jianping Sun, Gabriela Surdulescu, Jaana Suvisaari, Ionna Tachmazidou, Nicholas Timpson, Martin D. Tobin, Ana Valdes, Margriet Van Kogelenberg, Parthiban Vijayarangakannan, Peter M. Visscher, Louise V. Wain, Klaudia Walter, James T. R. Walters, Guangbiao Wang, Jun Wang, Yu Wang, Kirsten Ward, Elanor Wheeler, Tamieka Whyte, Hywel Williams, Kathleen A. Williamson, Crispian Wilson, Scott G. Wilson, Kim Wong, ChangJiang Xu, Jian Yang, Eleftheria Zeggini, Fend Zhang, Pingbo Zhang, Hou-Feng Zheng

**Affiliations:** 1 MRC Integrative Epidemiology Unit at the University of Bristol, University of Bristol, Oakfield House, Oakfield Grove, Bristol BS8 2BN, UK; 2 Department of Human Genetics, Wellcome Trust Sanger Institute, Genome Campus, Hinxton CB10 1HH, UK; 3 Department of Biomedical and Surgical Sciences, Ospedale Civile Maggiore, Azienda Ospedaliera-University of Verona, Verona, Italy; 4 Department of Haematology, University of Cambridge, Long Road, Cambridge CB2 0QQ, UK; 5 Centre for Cardiovascular Genetics, Institute of Cardiovascular Science, University College London, London WC1E 6JF, UK; 6 Wellcome Trust Centre for Human Genetics, University of Oxford, Oxford OX3 7BN, UK; 7 Department of Twin Research and Genetic Epidemiology, Kings College London, London SE1 7EH, UK; 8 MRC Epidemiology Unit, Institute of Metabolic Science, Addenbrooke’s Hospital, Box 285, Hills Road, Cambridge CB2 0SL, UK; 9 The Avon Longitudinal Study of Parents and Children, School of Social and Community Medicine, University of Bristol, Bristol BS8 2BN, UK; 10 D2K Research Group, School of Social and Community Medicine, University of Bristol, Oakfield House, Oakfield Grove, Bristol BS8 2BN, UK; 11 Horokopio University Athens, Eleftheriou Venizelou 70, Kallithea 176 76, Greece; 12 Division of Nephrology, Department of Internal Medicine and Medical Specialties, Catholic University, Largo Francesco Vito 1-00198, Rome, Italy; 13 Departments of Medicine, Human Genetics, Epidemiology and Biostatistics, Jewish General Hospital, 3755 Cote-Ste-Catherine Road, Montreal, Quebec, Canada H3T 1E2; 15 The Wellcome Trust Sanger Institute, Wellcome Trust Genome Campus, Hinxton, Cambridge CB10 1HH, UK; 16 Department of Pathology, King Abdulaziz Medical City, Riyadh, Saudi Arabia; 17 Department of Psychiatry, Trinity Centre for Health Sciences, St. James Hospital, James's Street, Dublin 8, Ireland; 18 Genetics and Genomic Medicine and Birth Defects Research Centre, UCL Institute of Child Health, London WC1N 1EH, UK; 19 Departments of Health Sciences and Genetics, University of Leicester, Leicester, UK; 20 Division of Developmental Disabilities, Department of Psychiatry, Queen's University, Kingston, Canada; 21 University of Cambridge Metabolic Research Laboratories, and NIHR Cambridge Biomedical Research Centre, Wellcome Trust-MRC Institute of Metabolic Science, Addenbrooke's Hospital, Cambridge CB2 0QQ, UK; 22 Department of Cardiovascular Medicine and Wellcome Trust Centre for Human Genetics, Roosevelt Drive, Oxford OX3 7BN, UK; 23 European Molecular Biology Laboratory, European Bioinformatics Institute, Wellcome Trust Genome Campus, Hinxton, Cambridge CB10 1 SD, UK; 24 Division of Psychiatry, The University of Edinburgh, Royal Edinburgh Hospital, Edinburgh EH10 5HF, UK; 25 Department of Medical Genetics, Cambridge Institute for Medical Research, University of Cambridge, Cambridge CB2 0XY, UK; 26 Institute of Psychiatry, Kings College London, 16 De Crespigny Park, London SE5 8AF, UK; 27 MRC Integrative Epidemiology Unit, School of Social and Community Medicine, University of Bristol, Oakfield House, Oakfield Grove, Clifton, Bristol BS8 2BN, UK; 28 NIHR BRC for Mental Health, Institute of Psychiatry and SLaM NHS Trust, King's College London, 16 De Crespigny Park, London SE5 8AF, UK; 29 Dubowitz Neuromuscular Centre, UCL Institute of Child Health and Great Ormond Street Hospital, London WC1N 1 EH, UK; 30 Department of Haematology, University of Cambridge, Long Road, Cambridge CB2 0PT, UK; 31 Department of Epidemiology, Biostatistics and Occupational Health, McGill University, Montreal, Quebec, Canada; 32 Institut für Humangenetik, Uniklinik Köln, Kerpener Str. 34, 50931 Köln, Germany; 33 The Department of Twin Research and Genetic Epidemiology, King's College London, St Thomas' Campus, Lambeth Palace Road, London SE1 7 EH, UK; 34 Social, Genetic and Developmental Psychiatry Centre, Institute of Psychiatry, King's College London, Denmark Hill, London SE5 8AF, UK; 35 Lilly Research Laboratories, Eli Lilly and Co. Ltd., Erl Wood Manor, Sunninghill Road, Windlesham, Surrey, UK; 36 MRC Centre for Neuropsychiatric Genetics and Genomics, Institute of Psychological Medicine and Clinical Neurosciences, School of Medicine, Cardiff University, Cardiff CF14 4XN, UK; 37 Sheffield Diagnostic Genetics Service, Sheffield Childrens' NHS Foundation Trust, Western Bank, Sheffield S10 2TH, UK; 38 University of Sussex, Brighton BN1 9RH, UK; 39 Sussex Partnership NHS Foundation Trust, Swandean, Arundel Road, Worthing, West Sussex BN13 3 EP, UK; 40 University College London (UCL), Molecular Psychiatry Laboratory, Division of Psychiatry, Gower Street, London WC1E 6BT, UK; 41 Computational Biology and Genomics, Biogen Idec, 14 Cambridge Center, Cambridge, Massachusetts 02142, USA; 42 Department of Medical and Molecular Genetics, Division of Genetics and Molecular Medicine, King's College London School of Medicine, Guy's Hospital, London SE1 9RT, UK; 43 BGI-Shenzhen, Shenzhen 518083, China; 44 University of Queensland Diamantina Institute, Translational Research Institute, Brisbane, Queensland, Australia; 45 MRC Human Genetics Unit, MRC Institute of Genetics and Molecular Medicine, at the University of Edinburgh, Western General Hospital, Edinburgh, EH4 2XU, UK; 46 The Genome Centre, John Vane Science Centre, Queen Mary, University of London, Charterhouse Square, London EC1M 6BQ, UK; 47 Cardiovascular Genetics, BHF Laboratories, Rayne Building, Institute Cardiovascular Sciences, University College London, London WC1E 6JJ, UK; 48 UCLA David Geffen School of Medicine, Los Angeles, California, USA; 49 Lady Davis Institute, Jewish General Hospital, Montreal, Quebec, Canada; 50 Departments of Medicine and Human Genetics, McGill University, Montreal, Quebec, Canada; 51 Department of Oncology, McGill University, Montreal, Quebec, Canada; 52 HeLEX—Centre for Health, Law and Emerging Technologies, Department of Public Health, University of Oxford, Old Road Campus, Oxford OX3 7LF, UK; 53 Department of Mathematical and Statistical Sciences, University of Colorado, Denver, Colorado 80202, USA; 54 Adaptive Biotechnologies Corporation, Seattle, Washington, USA; 55 Human Genetics Research Centre, St George's University of London, UK; 56 Department of Medicine and State Key Laboratory of Pharmaceutical Biotechnology, University of Hong Kong, 21 Sassoon Road, Hong Kong; 57 Behavioural and Brain Sciences Unit, UCL Institute of Child Health, London WC1N 1 EH, UK; 58 Analytic and Translational Genetics Unit, Massachusetts General Hospital, Boston, Massachusetts 02114, USA; 59 BGI-Europe, London; 60 National Institute for Health and Welfare (THL), Helsinki; 61 Wellcome Trust Centre for Human Genetics, Roosevelt Drive, Oxford OX3 7BN, UK; 62 Program in Medical and Population Genetics, Broad Institute of Harvard and MIT, Cambridge, Massachusetts 02132, USA; 63 Department of Statistics, University of Oxford, 1 South Parks Road, Oxford OX1 3TG, UK; 64 Department of Genetics, Harvard Medical School, Boston, Massachusetts 02115, USA; 65 The Patrick Wild Centre, The University of Edinburgh, Edinburgh EH10 5HF, UK; 66 The Department of Epidemiology and Biostatistics, Imperial College London, St. Mary's campus, Norfolk Place, Paddington, London W2 1PG, UK; 67 Department of Mathematics, Université de Québec À Montréal, Montréal, Québec, Canada; 68 Institute for Molecular Medicine Finland (FIMM), University of Helsinki, Helsinki, Finland; 69 Program in Medical and Population Genetics and Genetic Analysis Platform, The Broad Institute of MIT and Harvard, Cambridge, Massachusetts 02132, USA; 70 Institute of Neuroscience, Henry Wellcome Building for Neuroecology, Newcastle University, Framlington Place, Newcastle upon Tyne NE2 4HH, UK; 71 University of Helsinki, Department of Psychiatry, Helsinki; 72 MRC Epidemiology Unit, Institute of Metabolic Science, Box 285, Addenbrooke's Hospital, Hills Road, Cambridge CB2 0QQ, UK; 73 University College London (UCL) Genetics Institute (UGI) Gower Street, London WC1E 6BT, UK; 74 ALSPAC School of Social and Community Medicine, University of Bristol, Oakfield House, Oakfield Grove, Clifton, Bristol BS8 2BN, UK; 75 Institute of Medical Sciences, University of Aberdeen, AB25 2ZD, UK; 76 School of Oral and Dental Sciences, University of Bristol, Lower Maudlin Street, Bristol BS1 2LY, UK; 77 School of Experimental Psychology, University of Bristol, 12a Priory Road, Bristol BS8 1TU, UK; 78 Queensland Brain Institute, University of Queensland, Brisbane, Queensland 4072, Australia; 79 Department of Biology, University of Copenhagen, Ole Maaløes Vej 5, 2200 Copenhagen, Denmark; 80 Princess Al Jawhara Albrahim Center of Excellence in the Research of Hereditary Disorders, King Abdulaziz University, Jeddah, Saudi Arabia; 81 Macau University of Science and Technology, Avenida Wai long, Taipa, Macau 999078, China; 82 School of Medicine and Pharmacology, University of Western Australia, Perth, Western Australia, Australia; 83 Department of Endocrinology and Diabetes, Sir Charles Gairdner Hospital, Nedlands, Western Australia, Australia.

## Abstract

The analysis of rich catalogues of genetic variation from population-based sequencing provides an opportunity to screen for functional effects. Here we report a rare variant in *APOC3* (rs138326449-A, minor allele frequency ~0.25% (UK)) associated with plasma triglyceride (TG) levels (−1.43 s.d. (s.e.=0.27 per minor allele (*P*-value=8.0 × 10^−8^)) discovered in 3,202 individuals with low read-depth, whole-genome sequence. We replicate this in 12,831 participants from five additional samples of Northern and Southern European origin (−1.0 s.d. (s.e.=0.173), *P*-value=7.32 × 10^−9^). This is consistent with an effect between 0.5 and 1.5 mmol l^−1^ dependent on population. We show that a single predicted splice donor variant is responsible for association signals and is independent of known common variants. Analyses suggest an independent relationship between rs138326449 and high-density lipoprotein (HDL) levels. This represents one of the first examples of a rare, large effect variant identified from whole-genome sequencing at a population scale.

## Abstract (web summary)

Population-based genome sequencing provides an increasingly rich resource for the identification of low-frequency, large effect variants associated with clinically important phenotypes. Timpson *et al.* use UK10K data to identify a variant of the *APOC3* gene strongly associated with plasma triglyceride levels.

## Graphical abstract

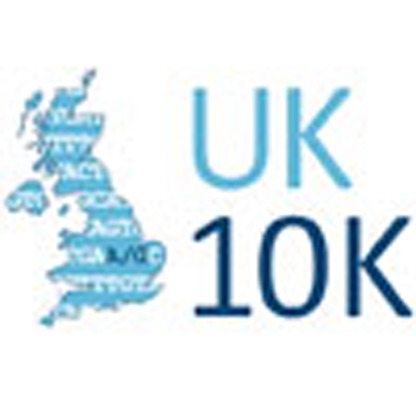

Lipid levels are heritable risk factors for coronary artery disease, and vascular outcomes and their therapeutic manipulation has well-characterized impacts on disease risk^1^. Genome-wide studies of common genetic variation and its contribution to commonly measured lipid moieties have been successful in identifying a large number of associated loci^2^,^3^; however, the aggregate contribution of all of these confirmed common variants accounts currently for only about 10–12% of the variation in low-density lipoprotein (LDL), high-density lipoprotein (HDL) and triglycerides (TGs)^3^. With current estimates of the heritability of these measures between 40% and 60%^4^, this leaves a considerable portion of variance unexplained. This may be contributed to by smaller common variant effects, as-yet undiscovered rare and potentially functional genetic variation, gene-by-gene interaction (epistasis) or by overestimates of heritability^5^. The availability of rich collections of variants through whole-genome sequencing (WGS) of well-phenotyped collections affords the unique opportunity to discover novel and potentially functional genetic variation associated with phenotypes of clinical interest.

Rare and highly penetrant variants identified in 24 different human genes have been identified through sequencing studies in families with rare monogenic lipid disorders^6^,^7^,^8^. Despite these studies, however, there has been little examination of these variants of low frequency (minor allele frequency (MAF)≤5%) on lipid profile at the level of the population. Studies that focus currently on exome content alone and have included variants of intermediate and low frequency have, however, reported larger genetic effects at lower MAF^9^,^10^,^11^. This type of work is relevant given that variants of large effect sizes have been suggested to segregate in populations at low frequencies under neutral or purifying models of evolution^12^. These genes and variants are likely to have considerable consequence on the health (expressed as odds ratios on the cardiovascular risk) for those who carry them, and may ultimately indicate novel therapeutic targets as already shown for *PCSK9* (ref. 13).

The UK10K Cohorts project ( http://www.uk10k.org/studies/cohorts.html) uses WGS to study the contribution of low-frequency and rare variation on a broad range of complex quantitative endpoints. Here we applied low read-depth WGS in individuals from two deeply phenotyped British cohorts, TwinsUK^14^ and the Avon Longitudinal Study of Parents and Children (ALSPAC)^15^ to analyse TG levels. Analyses revealed replicable evidence of a rare, functional, variant in the *APOC3* gene (rs138326449-A, MAF ~0.25% in the British population) strongly associated with plasma TG levels. This represents one of the first examples of a rare, large effect variant identified from WGS at a population based scale.

## Results

### Sequence data

A total of 3,910 individuals were sequenced to average 6.7 × mean read-depth using Illumina next-generation sequencing technology (Supplementary Methods ‘(Low read-depth WGS (cohorts data set)’). After applying stringent sample quality control filters, a total of 3,621 unrelated individuals of European ancestry (1,754 from TwinsUK and 1,867 from ALSPAC) were available for association. TG measurements were available for 3,202 individuals with sequence data, including 1,497 ALSPAC children (mean age 10 years, 50% females) and 1,705 TwinsUK adults, respectively (mean year 56 years, all females, Supplementary Table 1).

### Phenotypic association

To search the human genome for low-frequency and rare variants associated with TG levels, we first tested associations with 13,074,236 single-nucleotide variants (SNV) and 1,122,542 biallelic indels (MAF ≥0.1%) called from whole-genome-sequence data (Supplementary Table 2). Associations of TGs with genetic variation were tested in the ALSPAC and TwinsUK WGS data sets separately, and study-specific summary statistics were combined using inverse variance meta-analysis (Methods). There was no evidence for inflation of summary statistics in the combined sample (*λ*^(genomic control)^=0.99, Supplementary Fig. 1).

No variants gave evidence of association at conventional levels of genome-wide significance. However, four variants reached a second more exploratory tier of evidence for discovery in tests of association with TG (*P*-value ≤1 × 10^−7^) across the UK10K sample and were of interest as they mapped to a region around the *APOC3* locus on chromosome 11. Two of the variants found are common, rs964184-C (estimated allele frequency (EAF)=0.13%, *P*-value=6.81 × 10^−9^) and rs66505542-T (EAF=0.14%, *P*=1.87 × 10^−8^), in near-complete linkage disequilibrium (*r*^2^=0.90 in the UK10K sample) and have been previously associated with TG levels^2^,^3^.

**Figure 1:**
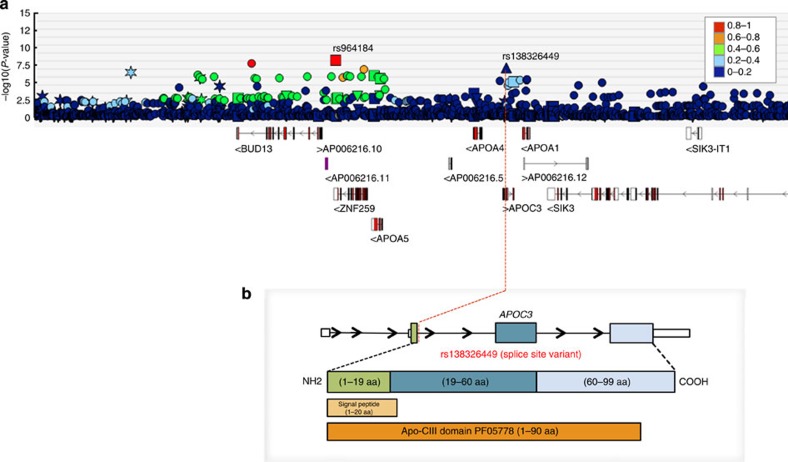
Regional plot of association between genetic variation at the ***APOC3*** **locus and plasma TG levels.** The figure is drawn using the UK10K Dalliance Browser. The tracks reported in **a** indicate (top to bottom): (i) *P*-value (on the –log10 scale) for association of SNPs in the *APOC3* region with TG levels. Symbols are coloured corresponding to *r*^2^ to indicate the extent of linkage disequilibrium of each SNP in the region with the index SNPs rs964184 (red square) and the splice variant rs138326449 (blue triangle) marked; (ii) GENCODE genes (from ftp://ngs.sanger.ac.uk/production/gencode/). (**b**) A cartoon illustrating the genic location of rs138326449 in the context of variable splicing of the *APOC3* gene.

**Figure 2:**
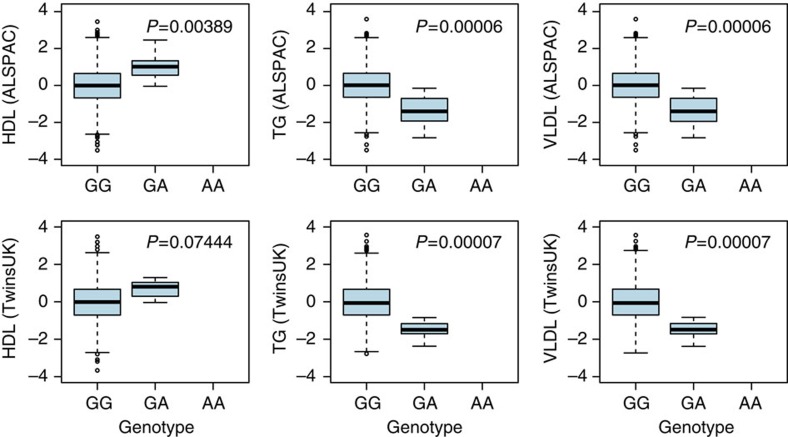
Association of lipid levels with rs138326449 at *APOC3.* Boxplots of associations between rs138326449 and TG, VLDL and HDL levels are shown as a function of carriage of allele A. Plots for HDL and TC are shown in Supplementary Fig. 2. *P* values indicate evidence for a linear relationship between lipid sub-fraction level and genotype (assuming an additive model). Box edges indicate the interquartile range (IQR; central line indicating the 50th centile) with the whisker indicating the lowest and highest daya still within 1.5 IQR of respective quartiles.

A third association meeting the nominal discovery threshold is a novel variant with low MAF, also mapping to the *APOC3* locus (Fig. 1). The rs138326449-A allele has an MAF of 0.25% and was associated with decreased TG levels corresponding to 1.43 (s.e=0.27) s.d. per allele (*P*-value=8.02 × 10^−8^) in the combined sample of 3,202 TwinsUK and ALSPAC participants with whole-genome sequence data (Fig. 2 and Table 1).

### Signal validation and refinement

We first validated whole-genome sequence-derived rs138326449 genotypes using overlapping genotype calls and showed perfect concordance in both TwinsUK and ALSPAC (Methods). We then took forward the variant for replication in five additional cohorts (*N*=12,831; Supplementary Table 1), where the variant was imputed with high accuracy (defined by imputation info values ≥0.4) using a novel reference panel obtained from combining UK10K data with data from the 1000 Genomes Project (Supplementary Methods). The rs138326449-A allele had similar allele frequency in the five additional cohorts, with the highest value observed in a population isolate from Greece (MAF=0.8%; Supplementary Table 1). The five cohorts provided independent replication of the association with decreased TG (−1.0 (s.e.=0.173) s.d., *P*-value=7.32 × 10^−9^, combined discovery and replication *P*-value=6.92 × 10^−15^), suggesting that this variant contributes to decreasing TG levels in multiple populations of Northern and Southern European origin, with similar effect sizes and allelic frequency.

We further tested association of the splice variant with the other three main lipid sub-fractions HDL, LDL and total cholesterol (TC), and with very-low-density lipoprotein (VLDL). The A allele at rs138326449 was associated with decreased VLDL levels in a combined sample of 7,891 participants with available data (−1.312 (s.e.=0.199), *P*-value=4.16 × 10^−11^) and with a moderate increase in HDL in 16,062 study participants (0.624 (s.e.=0.143), *P*-value=1.36 × 10^−5^; Table 1 and Fig. 2). Associations with LDL and TC were negligible (Supplementary Table 3 and Supplementary Fig. 2).

Analysis of the residual association between rs138326449 and TG levels conditional on other lipid-sub-fractions showed expected patterns in both children and adults when taking into account lipid-lowering drugs. Adjustment for VLDL removed the association between rs138326449 and TG levels. In the children of the ALSPAC study, there was also evidence of association between rs138326449 and HDL levels after adjustment for either TG or VLDL, which was also seen (although less strongly) in the 1958 British Cohort (Table 2).

### Further analysis of rare genetic variation

We next analysed the joint ALSPAC and TwinsUK sample with whole-genome sequence data to explore the contribution of common and rare genetic variants to TG associations in the *APOC3* region. We focused on a 640-kb recombination window containing the novel signal at rs138326449. Association between rs138326449 and TG was assessed conditioning simultaneously on known associated variants best tagging all previously published common variant signals at this locus (rs964184 and rs2075290 (refs 2, 3)) and other potentially novel independent loci derived from region-specific conditional analysis in the UK10K data. Other than rs138326449 and positive controls from previous studies, the only other potential independent signal single-nucleotide polymorphism (SNP) in this region was rs193204541, and neither did conditional analysis, including this variant, abolish evidence for association at rs138326449 (Supplementary Table 4) nor was this particular signal supported using available replication data.

We also examined the potential additional contribution of variants (frequency at or below 1%) by using Sequence Kernel Association Testing (SKAT^16^) in ~3-kb windows tiled over the *APOC3* region (Methods). Overall, seven windows had evidence for association with TG (*P*-value<1 × 10^−3^, equivalent to *P*-value=0.05 given a Bonferroni correction for multiple testing). The strongest of these was at chr11:116698501–116701500 (*P*-value=7.6 × 10^−7^). Despite specifically testing aggregates of rare variation, either one or a combination of the three independent SNP/SNV variants described above (that is, rs964184, rs2075290 and rs138326449) could account for six out of seven SKAT signals in this region (Supplementary Fig. 3). One region (chr11:116769001–116772000) showed nominal evidence of association with plasma TG levels (*P*-value=4 × 10^−4^) that could not be accounted for by association of any given individual SNV and that may represent a novel signal driven by multiple rare variants. Regional plots for all major lipid sub-fractions and SKAT results for this region can be found in Supplementary Figs 4 and 5). Results from a gene-based SKAT analysis across the *APOC3* gave greatest evidence for association with *APOC3* specifically; however, this region neither yielded results stronger than that shown from non-genic tiling nor were further regions implicated (Supplementary Table 5).

### Variance explained

Overall, in UK10K data across ALSPAC and TwinsUK, genetic variation in the *APOC3* region accounted for 2.71% (s.e.=1.39) of phenotypic variance in TG. This is in contrast to estimates of variance explained from the analysis of rs138326449 alone in children and adults not in the original discovery collections, which varied from 0.27 to 0.39% (Supplementary Table 6). Association results for known, TG-specific, positive controls are reported in Supplementary Table 7.

## Discussion

Within the cohorts arm of the UK10K study, we have collected low read-depth, whole genome sequence data and used this with a validation and replication panel to describe a rare SNV (MAF ~0.2% in Europeans) strongly associated with plasma TG. The variant rs138326449 accounts for single point and sequence kernel-based association signals at the known Mendelian locus *APOC3*, independently of known associations at this locus. We have replicated the association in up to 12,852 study participants from 5 additional population samples of Northern and Southern European origin, confirming this association, albeit at a more modest level (difference in plasma TG levels −1.0 s.d. (s.e.=0.173) per minor allele). The rare allele association with plasma TG level is consistent with an effect of between 0.5 and 1.5 mmol l^−1^ across children and adults dependent on population (Supplementary Fig. 6a). This is considerably larger than that reported in recent examinations of common variation and is one of the first of this nature to be reported from the use of population-based WGS. In context, the largest reported lipid effects from existing genome-wide association study (GWAS) are up to five times greater than that for the commonly recognized *FTO*rs9939609 variant and adult body mass index (which is ~0.01 s.d. change in body mass index); however, these are still more than 20 times lower than that seen here^2^,^17^. It is also notable that this effect is found in both children and adults, and in the presence or absence of lipid-altering interventions (Supplementary Fig. 6b).

The human *APOC3* gene is located in a gene cluster together with the *APOA1* and *APOA4* genes on the long arm of chromosome 11 (ref. 18). *APOC3* is expressed in the liver and intestine, and is controlled by positive and negative regulatory elements that are spread throughout the gene cluster^19^,^20^,^21^. There is considerable evidence to support the genetic contribution of this locus to hyperlipidaemia and, in particular, there have been correlations between apoCIII levels, plasma TG and VLDL TGs^22^,^23^. With this, the use of fibrates as a therapeutic intervention (known to reduce the apoCIII synthesis rate in humans^23^) has suggested that there is an important role for *APOC3* in TG metabolism. Moreover, transgenic mice expressing human apoCIII have shown that expression in the liver and intestine is correlated with elevated levels of VLDL TG, and where *apoE* is knocked out and *APOC3* expressed, huge accumulations of TG-rich VLDL can occur^24^,^25^.

The splice donor site reported here lies in a region of chromosome 11 previously shown to contain both common and rare variants affecting plasma TG levels. Restriction fragment length polymorphism variation within the non-coding part of exon 4 at this locus, haplotypic characterization of variation in the region and a single change within exon 3 of *APOC3* have all been related to either hypertriglyceridemia or familial combined hypercholesterolaemia^26^,^27^,^28^. More recently, a functional variant site (R19X) adjacent to rs138326449 and resulting in *APOC3* loss-of-function in homozygote carriers has been reported independently in two genetic isolates from the United States and Greece; however, this variant is very rare (EAF=0.05%) in the general European population and does not contribute to variance in TG in this study^29^,^30^. In each case, the impaired expression of *APOC3* is associated with reduced plasma TG levels and a coincident increase in HDL, in agreement with the inhibiting action of apoCIII on lipoprotein lipase. In the data here from UK10K, the total variance in plasma TGs explained by all genetic variation at this locus (down to and below 1% MAF) is ~2.7%. This is in comparison with this novel, low-frequency, genetic variant that accounts for somewhere between 0.27% and 0.39% of phenotypic variance.

The rs138326449 variant affects the essential di-nucleotide 5′-splicing site (GT to AT) of the first protein-coding exon of the protein-coding gene *APOC3*. The rare, TG-decreasing A allele is predicted to disrupt the correct splicing of the first protein-coding exon of *APOC3* (containing the Apo-CIII domain (PF05778) and a signal peptide (1–20 aa)), resulting in a marked change of the 5′-splicing site score (from 4.37 (G) to −3.81 (A))^31^ (Supplementary Fig. 7). Although it was not possible to validate the splicing event using existing liver expression atlas generated by the GTex project^32^ because of the lack of carriers in this data set, we note that this position is highly conserved (phastCons=0.996, a measurement of evolutionary conservation based on multiple alignments of 100 vertebrates) through vertebrates^33^, supporting a probably potential, functional consequence for this site.

Recognized within the Adult Treatment Panel III and as part of the definition of the metabolic syndrome ( https://www.nhlbi.nih.gov/health-pro/guidelines/current/cholesterol-guidelines/index.htm), TG and TG-rich remnants are probably risk factors in cardiovascular disease^34^. Meta-analysis of 17 prospective studies has suggested that TGs are independent contributors to coronary heart disease risk and data from both the Münster Heart and Caerphilly studies have supported this^35^,^36^. This effect appears to be present independent of LDL-cholesterol and HDL-cholesterol levels^37^,^38^; however, these findings are not simple in interpretation. The current largest meta-analysis based on the same phenotypes has shown contradictory results^39^ and, in addition, an outstanding issue in these analyses remains the difficulty in assessing the independent impact of reduced or elevated TG levels from HDL. In our data, rs138326449 is associated with reduced TG in line with predicted lower levels of functional apoCIII in carriers of the A allele. However, this effect is not unique to TG levels, with a coincident and independent association with HDL making the interpretation of downstream effects of this variant (or variants at this locus exerting a similar effect) difficult in terms of causal inference.

In the absence of a clear explanation for the complex relationship between apolipoprotein gene effects and multiple lipid outcomes, the notion of overall lipid profile as a risk factor may be the most acceptable paradigm. To this end, variants such as rs138326449 do potentially provide information about the impact of interventions aimed at changing lipid profile. In this context, the impact of *APOC3* inhibition through approaches such as targeted antisense oligonucleotide use^40^ can be modelled given observations such as that made in this study. This essentially represents an applied Mendelian randomization^41^ experiment, and with coincident disease status available, this type of study may help identify future, gene-targeted, therapeutic interventions.

## Methods

### ALSPAC WGS discovery sample

The ALSPAC is a long-term health research project. More than 14,000 mothers enrolled during pregnancy in 1991 and 1992, and the health and development of their children has been followed in great detail ever since. A random sample of 2,040 study participants was selected for WGS. The ALSPAC Executive Committee approved the study and all participants gave signed consent to the study.

Non-fasting plasma levels of TC, HDL and TG at age 9 years were measured with enzymatic colorimetric assays (Roche) on a Hitachi Modular P Analyser. HDL, TGs and TC (all in mmol l^−1^) were measured as described previously^42^. LDL was derived from the Friedwald formula: TC-(HDL Cholesterol+(TG/2.19))^43^. We calculated VLDL as VLDL Cholesterol (mmol l^−1^)=TC-LDL Cholesterol–HDL Cholesterol.

### TwinsUK WGS discovery sample

TwinsUK is a nation-wide registry of volunteer twins in the United Kingdom, with about 12,000 registered twins (83% female, equal number of monozygotic and dizygotic twins, predominantly middle-aged and older). Over the last 20 years, questionnaire and blood/urine/tissue samples have been collected for over 7,000 subjects, as well as three comprehensive phenotyping assessments. The primary focus of study has been the genetic basis of healthy aging process and complex diseases, including cardiovascular, metabolic, musculoskeletal and ophthalmologic disorders. Alongside the detailed clinical, biochemical, behavioural and socio-economic characterization of the study population, the major strength of TwinsUK is availability of several ‘omics’ technologies for the participants. The database was used to study the genetic and environmental aetiology of age-related complex traits and diseases. The St Thomas’s Hospital Ethics Committee approved the study and all participants gave signed consent to the study.

Enzymatic colorimetric assays were used to measure serum levels of TC, HDL and TGs, and were measured using three analysing devices (Cobas Fara; Roche Diagnostics, Lewes, UK; Kodak Ektachem dry chemistry analysers (Johnson and Johnson Vitros Ektachem machine, Beckman LX20 analysers, Roche P800 modular system)). The majority of discovery samples were fasted before measurement (96%).

*Low read-depth WGS (cohorts data set)*. Low read-depth WGS was performed at both the Wellcome Trust Sanger Institute and the Beijing Genomics Institute (BGI). DNA (1–3 μg) was sheared to 100–1,000 bp using a Covaris E210 or LE220 (Covaris, Woburn, MA, USA). Sheared DNA was subjected to Illumina paired-end DNA library preparation. Following size selection (300–500 bp insert size), DNA libraries were sequenced using the Illumina HiSeq platform as paired-end 100 base reads according to the manufacturer’s protocol.

### Alignment and BAM processing

Data generated at the Sanger Institute and BGI were aligned to the human reference separately by the respective centres. The BAM files produced from these alignments were submitted to the European Genome-phenome Archive. The Vertebrate Resequencing Group at the Sanger Institute then performed further processing.

### Alignment

Sequencing reads that failed quality control (QC) were removed using the Illumina GA Pipeline, and the rest were aligned to the GRCh37 human reference, specifically the reference used in Phase 1 of the 1000 Genomes Project ( ftp://ftp.1000genomes.ebi.ac.uk/vol1/ftp/technical/reference/human_g1k_v37.fasta.gz). Reads were aligned using BWA (v0.5.9-r16)^44^.

### BAM improvement and sample file production

Further processing to improve SNV and INDEL calling, including realignment around known INDELs, base quality score recalibration, addition of BAQ tags, merging and duplicate marking follows that used for Illumina low-coverage data in Phase 1 of the 1000 Genomes Project. Software versions used for UK10K for the steps described in that section were GATK version 1.1-5-g6f43284, Picard version 1.64 and samtools version 0.1.16.

### Variant calling

SNV and INDEL calls were made using samtools/bcftools (version 0.1.18-r579)^45^ by pooling the alignments from 3,910 individual low read-depth BAM files. All-samples and all-sites genotype likelihood files (bcf) were created with samtools mpileup.

### INDEL pre-filtering

The observation of spikes in the insertion/deletion ratio in sequencing cycles of a subset of the sequencing runs were linked to the appearance of bubbles in the flow cell during sequencing. To counteract this, the bamcheck utility from the samtools package was used to create a distribution of INDELs per sequencing cycle. Lanes with INDELs predominantly clustered at certain read cycles were marked as problematic (159 samples). In the next step, we checked mapped positions of the affected reads to see whether they overlapped with called INDELs, which they did for 1,694,630 called sites. The genotypes and genotype likelihoods of affected samples were then set to the reference genotype unless there was a support for the INDEL also in a different, unaffected lane from the same sample. In total, 140,163 genotypes were set back to reference and 135,647 sites were excluded by this procedure. Note that this step was carried out on raw, unfiltered calls before Variant Quality Score Recalibration filtering.

### Site filtering

Variant Quality Score Recalibration^46^ was used to filter sites. For SNVs, the GATK (version 1.3–21) UnifiedGenotyper was used to recall the sites/alleles discovered by samtools to generate annotations to be used for recalibration. Recalibration for the INDELs used annotations derived from the built-in samtools annotations. The GATK VariantRecalibrator was then used to model the variants, followed by GATK ApplyRecalibration, which assigns VQSLOD (variant quality score log odds ratio) values to the variants. For SNV sites, a truth (GRCh37) sensitivity of 99.5%, which corresponded to a minimum VQSLOD score of −0.6804 was selected; that is, for this threshold, 99.5% of truth sites were retained. For INDEL sites, a truth sensitivity of 97%, which corresponded to a minimum VQSLOD score of 0.5939 was chosen. Finally, we also introduced the filter *P*<10^−6^ to remove sites that failed the Hardy–Weinberg equilibrium (302,388 sites removed) and removed sites with evidence for differential frequency (logistic regression *P*-value>1e−2) between samples sequenced at BGI and Wellcome Trust Sanger Institute (277,563 sites removed).

Given the presence of structure by genotyping batch, we ran a genome-wide association analysis for the binary variable ‘sequencing centre’ (‘BGI’/‘SANGER’) using a logistic regression model. SNPs (335,982) were associated with batch at a conservative threshold of *P*-value≤0.01 and formed a list that were subsequently filtered out from the genotype set, removing the batch effect due to sequencing centre.

### Post-genotyping sample QC

Of the 4,030 samples (1,990 TwinsUK and 2,040 ALSPAC) that were submitted for sequencing, 3,910 samples (1,934 TwinsUK and 1,976 ALSPAC) were sequenced and went through the variant calling procedure. Low-quality samples were identified before the genotype refinement by comparing the samples with their GWAS genotypes using ~20,000 sites on chromosome 20 (see Supplementary Methods for full details).

### Genotype refinement

The missing and low-confidence genotypes in the filtered VCFs were refined out through an imputation procedure with BEAGLE 4, rev909 (ref. 47). The programme was run with default parameters (see Supplementary Methods for full details). After imputation, chunks were recombined using the vcf-phased-join script from the vcftools [vcftools] package.

### Post-refinement sample QC

Additional sample-level QC steps were carried out on refined genotypes, leading to the exclusion of additional 17 samples (16 TwinsUK and 1 ALSPAC) because of one or more of the following causes: (i) non-reference discordance with GWAS SNV data>5% (12 TwinsUK and 1 ALSPAC), (ii) multiple relations to other samples (13 TwinsUK and 1 ALSPAC) or (iii) failed sex check (3 TwinsUK and 0 ALSPAC).

To exclude the presence of participants of non-European ancestry in our data set, we merged a pruned data set to the 11 HapMap3 populations^48^ and performed a principal components analysis using EIGENSTRAT^49^. A total of 44 participants (12 TwinsUK and 32 ALSPAC) did not cluster to the European (CEU) cluster of samples and were removed from association analyses.

The final sequence data set that was used for the association analyses comprises 3,621 samples (1,754 TwinsUK and 1,867 ALSPAC).

### Re-phasing

SHAPEIT2 (ref. 50) was then used to rephase the genotype data. The VCF files were converted to binary ped format. Multiallelic and MAF<0.02% (singleton and monomorphic) sites were removed. Files were then split into 3-mbp chunks with ±250 kbp flanking regions. SHAPEIT (v2.r727) was used to rephase the haplotypes.

### Imputation from the combined UK10K+1000 Genomes Panel

For each of the cohorts, we had additional GWA data available. For ALSPAC, 6,557 samples were measured on Illumina HumanHap550 arrays and passed QC (population stratification, sex check, heterozygosity and relatedness (identity by state (IBS)>0.125)). For TwinsUK, 2,575 samples were genotyped on Illumina HumanHap300 or Illumina Human610 arrays. These samples passed QC on relatedness (IBS>0.125), population stratification, heterozygosity, zygosity and sex checks. Samples from the imputed data sets were unrelated to the sequence data sets (IBS>0.125). Variants discovered through WGS of the TwinsUK and ALSPAC cohorts were used for the development and use of a reference panel for imputation within the TwinsUK and ALSPAC GWA data sets. In other collections, these along with variants known from 1000 Genomes were imputed increasing the sample size for single point association analysis to 12,724 subjects. We developed new functionality in IMPUTE2 (ref. 51) that uses each reference panel to impute the missing variants in its counterpart, and then combine the two reference panels at the union set of sites. We tested the 3 reference panels for imputing 3 SNP array data, a sub-sample of 1,000 individuals from the UK10K WGS data set, 4 European samples (3 CEU, 1 TSI) sequenced by Complete Genomics (depth: 80 × )^52^ and an Italian isolate genotyped on core-exome SNP array (see Supplementary Methods for full details).

### Validation genotyping

For ALSPAC, the entire cohort (10,145 participants, including 38 carriers of the rare A allele) was genotyped using KASP at KBioscience ( www.lgcgenomics.com/; see Supplementary Methods for full details). For TwinsUK, genotyping accuracy was evaluated against a data set comprising ~250 high-coverage exomes sequenced in overlapping samples^53^. Of the six carriers detected in our study, four were overlapping and correctly called also in the exome data set, yielding a genotyping accuracy of 100%. There was 100% concordance with the genotypes called from the whole-genome data set.

### Trait standardization

Each cohort applied a standardized protocol for preparation of phenotypes, as follows. Female and male participants were divided into separate groups and TwinsUK participants were further divided into two unrelated subsets. Outliers deviating ≥4 or 5 s.d. (depending on the study) from the sample mean for a given trait were excluded from analysis (for this step, TGs were log transformed). The filtering of TG data by extremes of phenotype does not have a substantive impact on the numbers of rare variant carriers in this data set (although overall there is likely to be an enrichment for rare variant carriage in at the low end of the TG distribution in large collections). To approximate normality, each data set was inverse normal rank transformed in each group separately, and residuals were further computed by adjustment for age and age^54^ squared as a fixed effect. In TwinsUK, analyser effects were computed additionally as a random effect if associated with phenotype. Finally, residuals were standardized before combining males and females. In ALSPAC, trait residuals were computed jointly from the WGS and GWA samples. Details of trait transformation and statistical methods applied in each study are summarized in Supplementary Table 2. For conditional lipid analyses (Supplementary Table 4), all lipid sub-fractions and TC were inverse rank transformed before analyses. Pearson’s correlation coefficients were used to assess the correlation between variables, and linear regression was used to assess the relationship between variation at rs138326449 and lipid sub-fraction having serially conditioned on other lipids. For main analyses, where VLDL was missing from replication collections, it was not included given the correlation between TG and VLDL when derived from TC, HDL and LDL. This is illustrated where for regional analyses across ALSPAC and the 1958 British Cohort VLDL is calculated for purposes of illustration (Table 2).

### Associations between lipids and SNVs and indels

We assessed associations between 14,196,778 genetic variants (13,074,236 SNPs and 1,122,542 biallelic indels, MAF ≥0.1%) and lipid traits (LDL, HDL, TG, TC and VLDL) calculated as described before, using linear regression models assuming additive genetic models. For primary analyses, associations were tested using a genotype dosage-based test implemented in the SNPTESTv4.2 software package^54^, apart for the TwinsUK GWAS and HELIC MANOLIS data sets, where mixed linear models were used to account for family structure using the GEMMA software^55^.

### Meta-analysis of associations with SNVs

Summary statistics from individual studies were combined using fixed-effect inverse variance meta-analysis implemented in GWAMA v2.1 (ref. 56).

### Region-specific analyses

Conditional analyses of genotype and rare variant aggregate association were undertaken within a joint sample from the UK10K cohort WGS data set (ALSPAC and TwinsUK) adjusting for study origin by residualizing transformed TG on an indicator variable for study. Records of region-specific recombination used to derive the recombination interval boundaries were retrieved from ( http://hapmap.ncbi.nlm.nih.gov/downloads/recombination/2011-01_phaseII_B37/), and this analysis was limited to a 640-kb window of chromosome 11 marked by a recombination fraction <25%. Evidence for previous genetic associations was available from existing studies^2^,^3^ and best tag variants for positive controls were derived by using PLINK to assess linkage disequilibrium across positive controls^57^. Evidence for further novel independent TG associations across the *APOC3* region in this data set were assessed using GCTA^58^. We considered all SNPs and bi-allelic indels seen at least twice that had any evidence of association with TG (*P*<1 × 10^−3^) alongside those previously associated with lipid levels irrespective of association result in this sample^58^,^60^. Conditional analyses were undertaken for rs138326449 given all potentially independent contributing loci in this region using GCTA. GCTA was also used to calculate the total genetic contribution to variance in TG for the same region having calculated a matric of relatedness from the whole of chromosome 11. In addition to regional analyses, estimates of variance explained for rs138326449 alone were derived from the ALSPAC and 1958 birth cohort collections using linear regression taking into account the covariables age, age, sex and lipid-lowering drugs in the case of the 1958 birth cohort. Analyses for this were undertaken using STATA version 13 (StataCorp. 2013. Stata Statistical Software: Release 13; StataCorp LP, College Station, TX).

SKAT^16^ was undertaken across the *APOC3* region. Sequence-derived genotypes with MAF capped at 1% were extracted and split into sub-regions containing as close to 50 variants as possible. These were analysed using SKAT and all signals with evidence for association *P*-value<1 × 10^−3^, equivalent to *P*-value=0.05 given a Bonferroni correction for multiple testing across this region, were taken forward for further analyses. We then re-formulated phenotype-containing fam files for SKAT analysis having conditioned sequentially on known positive control or novel independent contributing SNPs in the region before re-running SKAT analyses. Results from a gene-based SKAT analysis were generated by running SKAT (again with MAF ≤1%) for genes contained within the *APOC3* region. Genes for this analysis were defined by GENCODE (v15) within positions 115,820,914 and 117,103,241 on chromosome 11. In more detail, variants within exons and splice variants were tested in windows up to 51 variants per window. If there were >50 exonic and splice variants per gene, then variants were split in two ways: first by combining neighbouring exons so that the number of variants was about evenly split between windows, and second by tiling across the concatenated exons with maximal 51 variants per window but starting halfway the first window that was generated by the first approach.

### Replication samples

Description of the replication samples is given in the Supplementary Methods.

## Disclaimer

This publication is the work of the authors and N.J.T., G.D.S. and S.R. will serve as guarantors for the contents of this paper. N.J.T. and G.D.S. work within a MRC unit at the University of Bristol. Please note that the ALSPAC website contains details of all the data that is available through a fully searchable data dictionary ( www.bris.ac.uk/alspac/researchers/data-access/data-dictionary). T.D.S. is holder of an ERC Advanced Principal Investigator award.

## Additional information

**How to cite this article:** Timpson, N. J. *et al.* A rare variant in *APOC3* is associated with plasma triglyceride and VLDL levels in Europeans. *Nat. Commun.* 5:4871 doi: 10.1038/ncomms5871 (2014).

## Supplementary Material

Supplementary Information Supplementary Figures 1-7, Supplementary Tables 1-7, Supplementary Methods and Supplementary References

## Figures and Tables

**Table 1:** Summary of genetic associations between rs138326449 and levels of TG, VLDL and HDL in discovery and replication sample sets.

**Sample**	**Metric**	**TG**	**HDL**
TwinsUK WGS	Beta (s.e.)	−0.60 (0.23)	0.328 (0.18)
(EAF 0.23%)	*P*-value	7.7 × 10^−3^	0.06
	*N*	1,705	1,713
	Info metric	0.78	0.78
			
ALSPAC WGS	Beta (s.e.)	−0.52 (0.20)	0.33 (0.11)
(EAF 0.28%)	*P*-value	9.3 × 10^−3^	3.4 × 10^−3^
	*N*	1,497	1,497
	Info metric	0.94	0.94
			
Discovery combined	Beta (s.e.)	−1.43 (0.27)	0.84 (0.27)
	*P*-value	8.0 × 10^−8^	2.1 × 10^−3^
	*N*	3,202	3,210
			
1958BC (EAF 0.15%)	Beta (s.e.)	−1.35 (0.33)	1.04 (0.32)
	*P*-value	4.3 × 10^−5^	1.2 × 10^−3^
	*N*	5,485	5,493
	Info metric	0.55	0.55
			
INCIPE (EAF 0.26%)	Beta (s.e.)	−0.93 (0.43)	0.63 (0.42)
	*P*-value	0.03	0.13
	*N*	1,382	1,382
	Info metric	0.78	0.78
			
TwinsUK GWAS	Beta (s.e.)	−0.90 (0.36)	0.79 (0.34)
(EAF 0.29%)	*P*-value	0.01	0.02
	*N*	1,882	1,896
	Info metric	0.75	0.75
			
ALSPAC GWAS	Beta (s.e.)	−1.83 (0.56)	1.30 (0.55)
(EAF 0.2%)	*P*-value	1.2 × 10^−3^	0.02
	*N*	2,820	2,820
	Info metric	0.77	0.77
			
HELIC M (EAF 0.78%)	Beta (s.e.)	−1.26 (0.36)	0.74 (0.36)
	*P*-value	5.4 × 10^−4^	0.04
	*N*	1262	1264
	Info metric	0.42	0.42
			
Combined replication	Beta (s.e.)	−1.00 (0.17)	0.54 (0.17)
	*P*-value	7.3 × 10^−9^	1.3 × 10^−3^
	*N*	12,831	12,855
			
Overall	Beta (s.e.)	−1.13 (0.15)	0.62 (0.14)
	*P*-value	6.9 × 10^−15^	1.4 × 10^−5^
	*N*	16,033	16,065

ALSPAC, Avon Longitudinal Study of Parents and Children; EAF, estimated allele frequency; GWAS, genome-wide association study; HDL, high-density lipoprotein; TG, triglyceride; VLDL, very low-density lipoprotein.

Data is reported for the discovery sample of TwinsUK and ALSPAC whole-genome sequence, and for the five replication samples where the variant was imputed. For each trait, the Beta (s.e.) is expressed in s.d. units for the population distribution of the corresponding trait. Beta reports a standardized per allele effect and Info metric reports the ‘proper info’ from the imputation process.

**Table 2:**
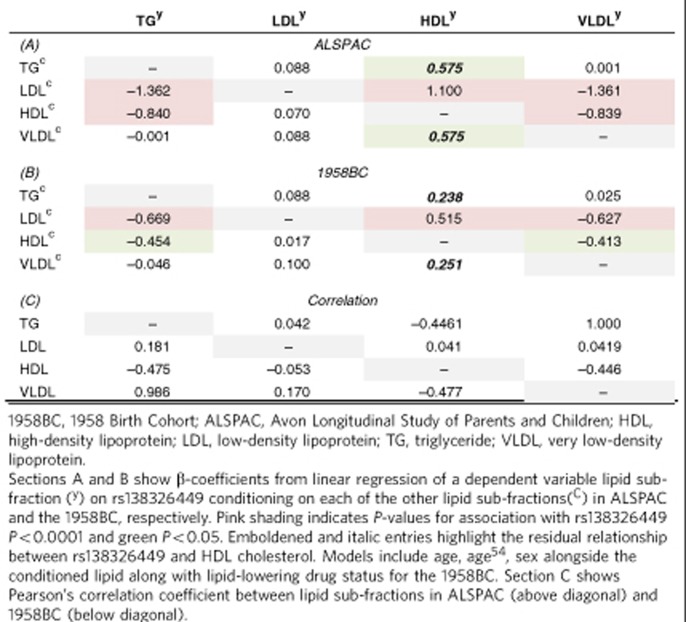
Conditional associations between rs138326449 and lipid sub-fraction in the ALSAPC and the 1958BC.
