# Supplementary material for: A rare variant in APOC3 is associated with plasma triglyceride and VLDL levels in Europeans
Source: Nat Commun. 2014 Sep 16;5:4871. doi: 10.1038/ncomms5871 (PMC4167609; doi:10.1038/ncomms5871)
Supplement: Supplementary Information — Supplementary Figures 1-7, Supplementary Tables 1-7, Supplementary Methods and Supplementary References [file ncomms5871-s1.pdf]

Supplementary Figure 1. QQ plot for association of SNPs with TG levels in the combined analysis of the two whole-genome sequence datasets.

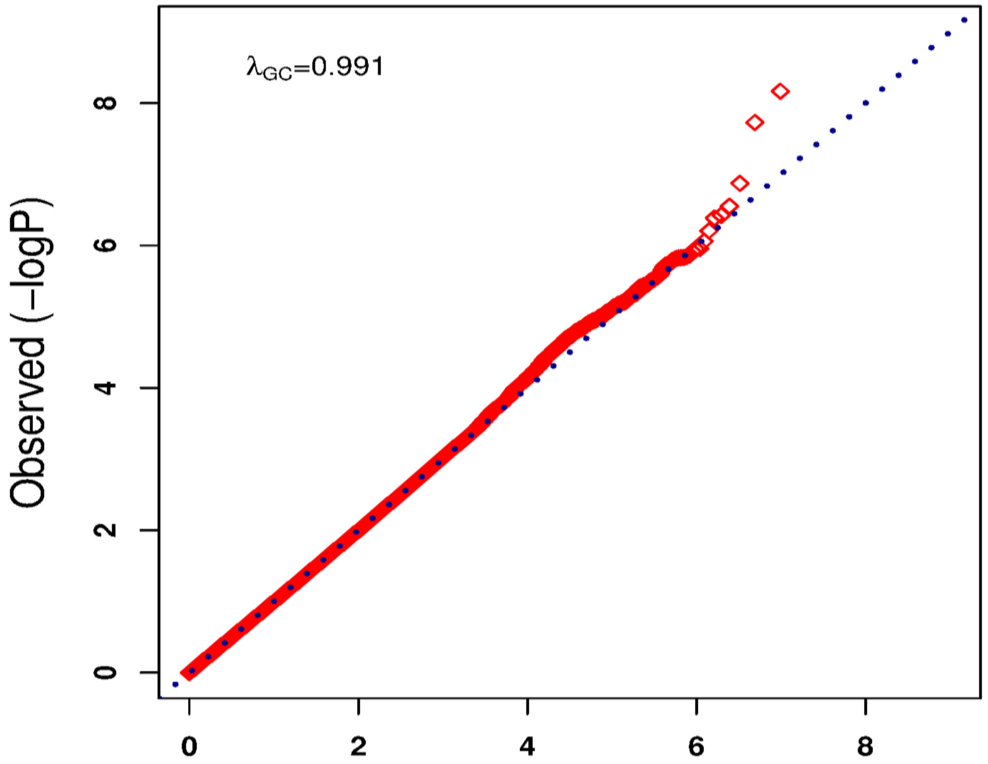

Red diamonds indicate observed p-values plotted against those expected from an empirically derived null distribution.

Supplementary Figure 2. Association of lipid levels with rs138326449 at *APOC3*. Boxplots of associations between rs138326449 and total cholesterol (TC), and low-density lipoprotein (LDL) levels are shown as a function of carriage of allele A.

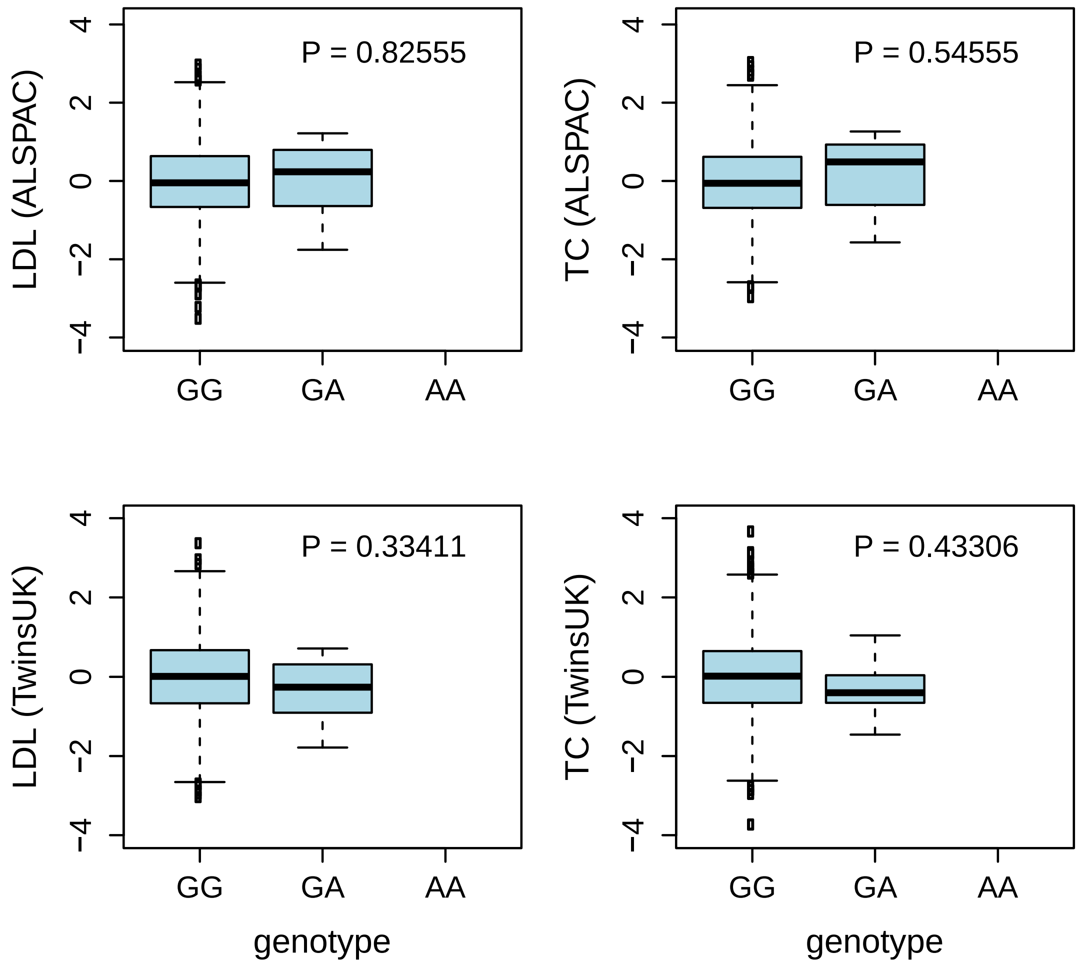

Supplementary Figure 3. Regional plot of both sequence kernel and single point tests for association between genetic variation and circulating TG.

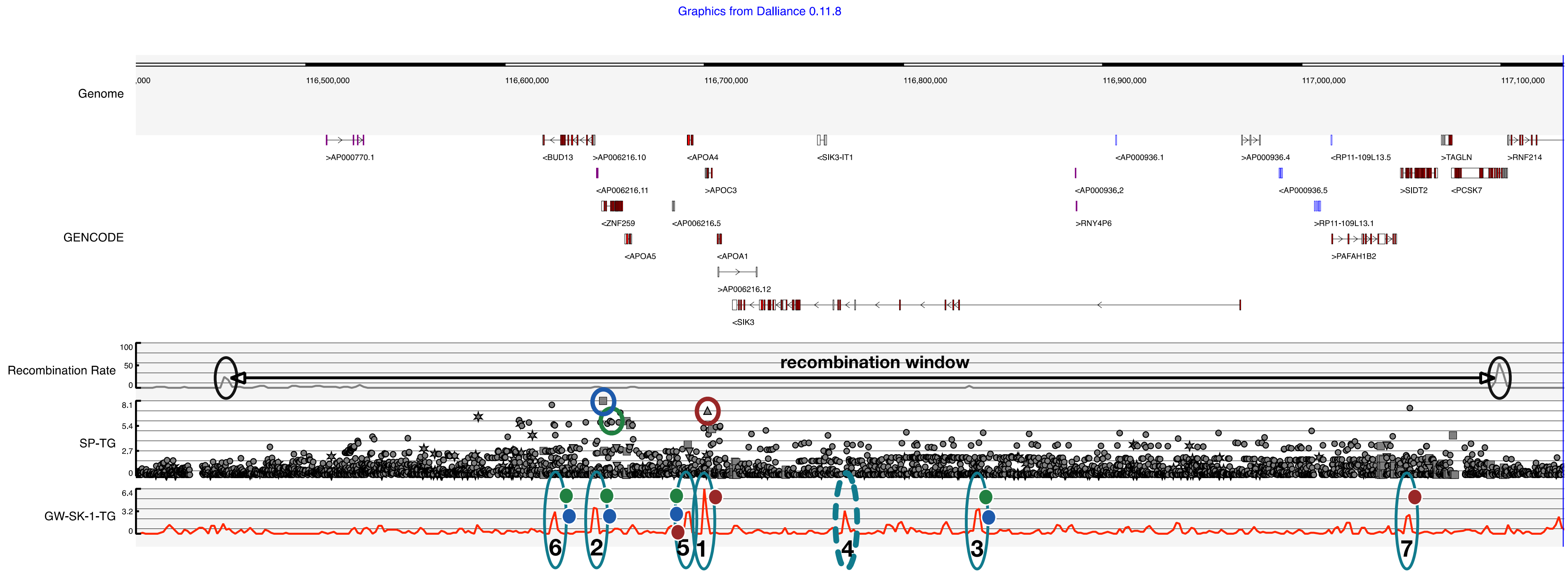

Tracks follow: Genomic position (bp), GENCODE gene annotation, local recombination rate, SP-TG (singlepoint association results for triglyceride, inverse log10 p values from a meta-analysis across ALSPAC and TUK within UK10K), GW-SK-1-TG (sequence kernel association test association results for triglyceride, inverse log10 p values from a meta-analysis across ALSPAC and TUK within UK10K). "recombination window" indicates the APOC3 region defined as all within a recombination fraction <25%). Small open circles highlight singlepoint association results for the three independent positive controls or novel independent signals across the *APOC3* region (blue=rs964184, green=rs2075290, red =rs138326449). Ovals highlight 7 (1-7 labelled by decreasing evidence for association) GW-SK-1-TG results with any evidence for association ($p<1x10^{-3}$). Closed circles with colours corresponding to specific singlepoint results indicate which single nucleotide polymorphisms are able to account for which sequence kernel association test). Broken oval highlights one sequence kernel association test result which cannot be explained completely by singlepoint results.

Supplementary Figure 4. Regional plot of single point tests for association between genetic variation and circulating measures of all major lipid sub-fractions.

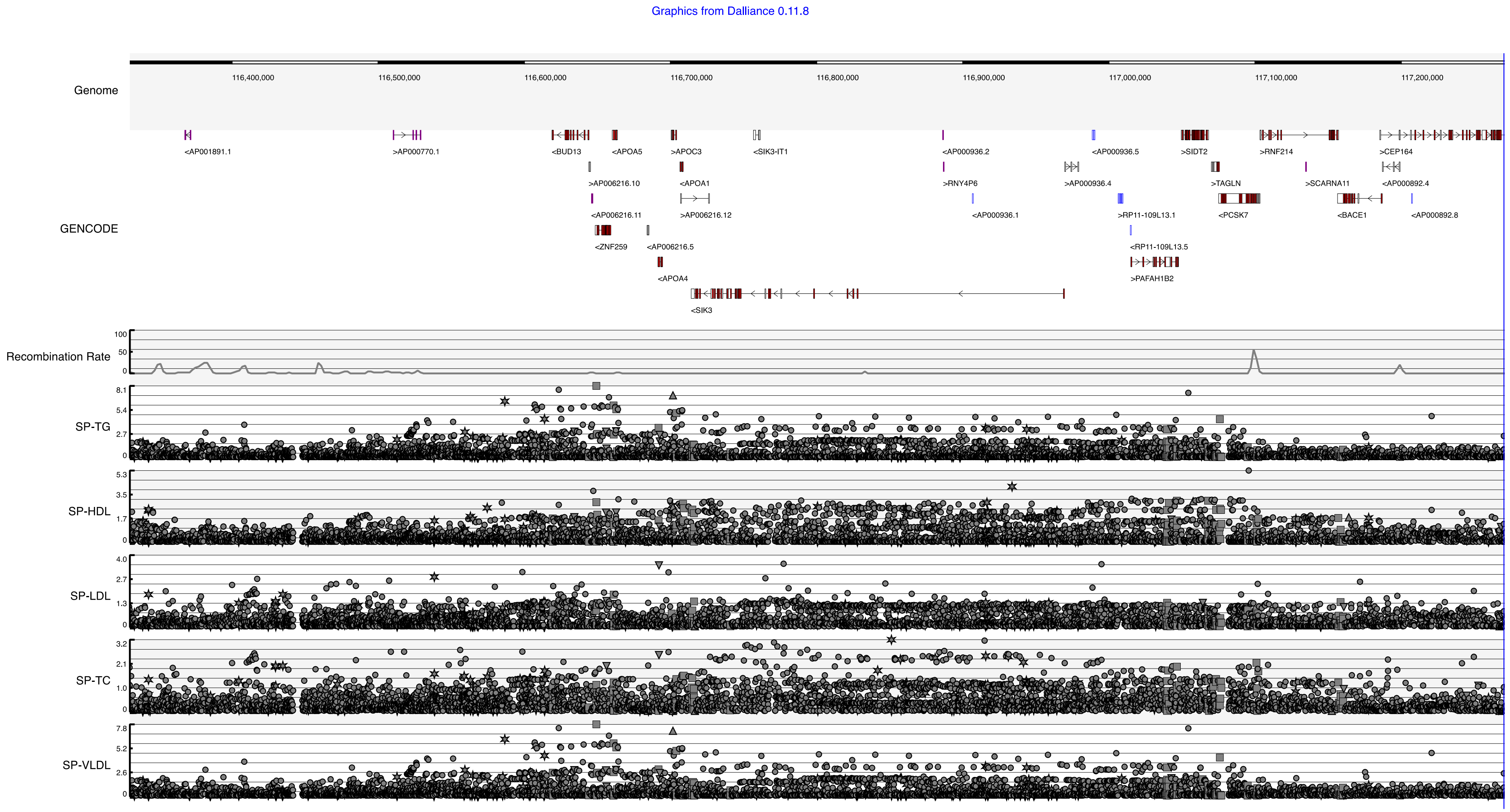

Tracks follow: as per **Supplementary Figure 2**, but only with single point results and across all major lipid sub-fractions (SP-HDL singlepoint association results for HDL, SP-LDL singlepoint association results for HDL, SP-VLDL singlepoint association results for VLDL, SP-TC singlepoint association results for total cholesterol).

Supplementary Figure 5. Regional plot of rare variant tests for association between genetic variation and circulating measures of all major lipid sub-fractions.

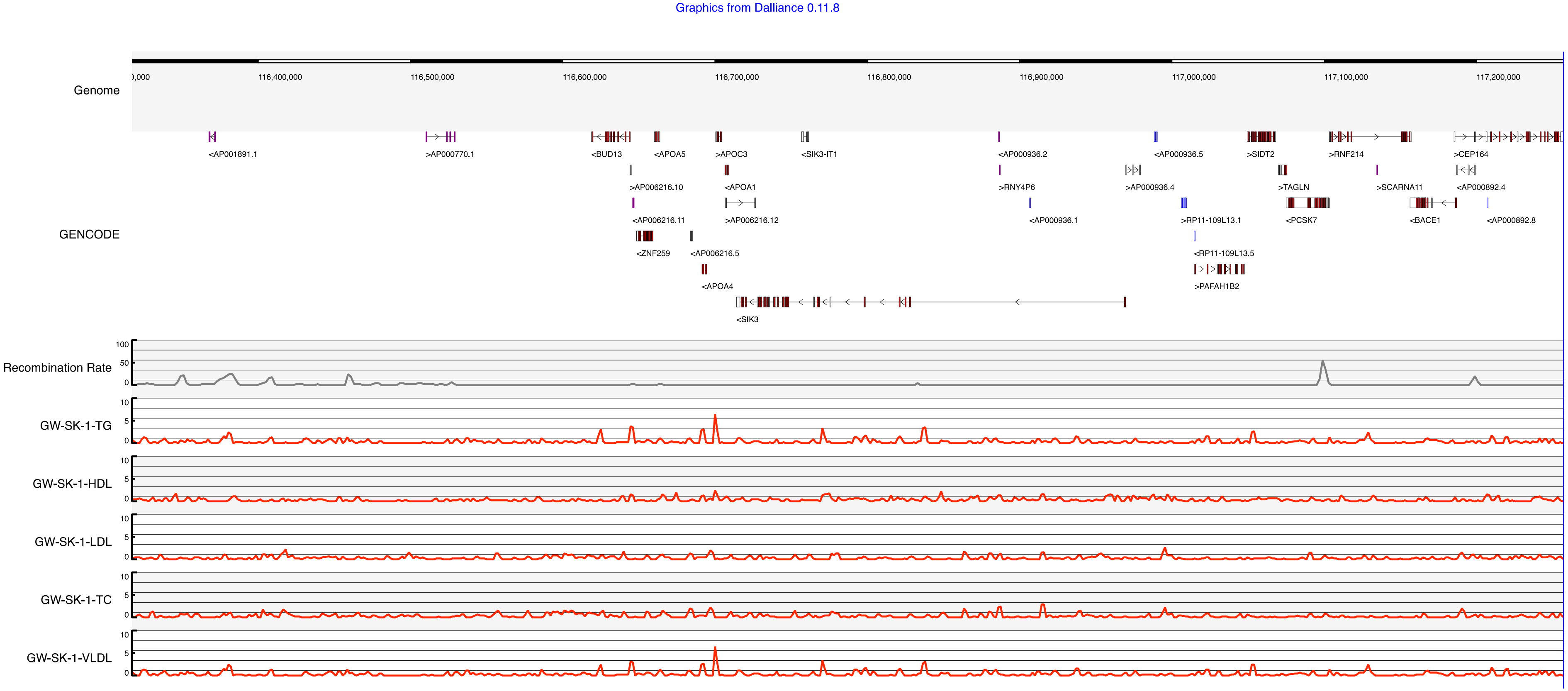

Tracks follow: as per **Supplementary Figure 2**, but only with sequence kernel association test results and across all major lipid sub-fractions (SK-HDL SKAT association results for HDL, SK-LDL SKAT association results for HDL, SK-VLDL SKAT association results for VLDL, SK-TC SKAT association results for total cholesterol).

Supplementary Figure 6. Distribution of log transformed and native triglyceride levels with reference to both adult thresholds for hypertriglyceridaemia and HDL levels in children from the ALSPAC study and adults from the 1958 birth cohort.

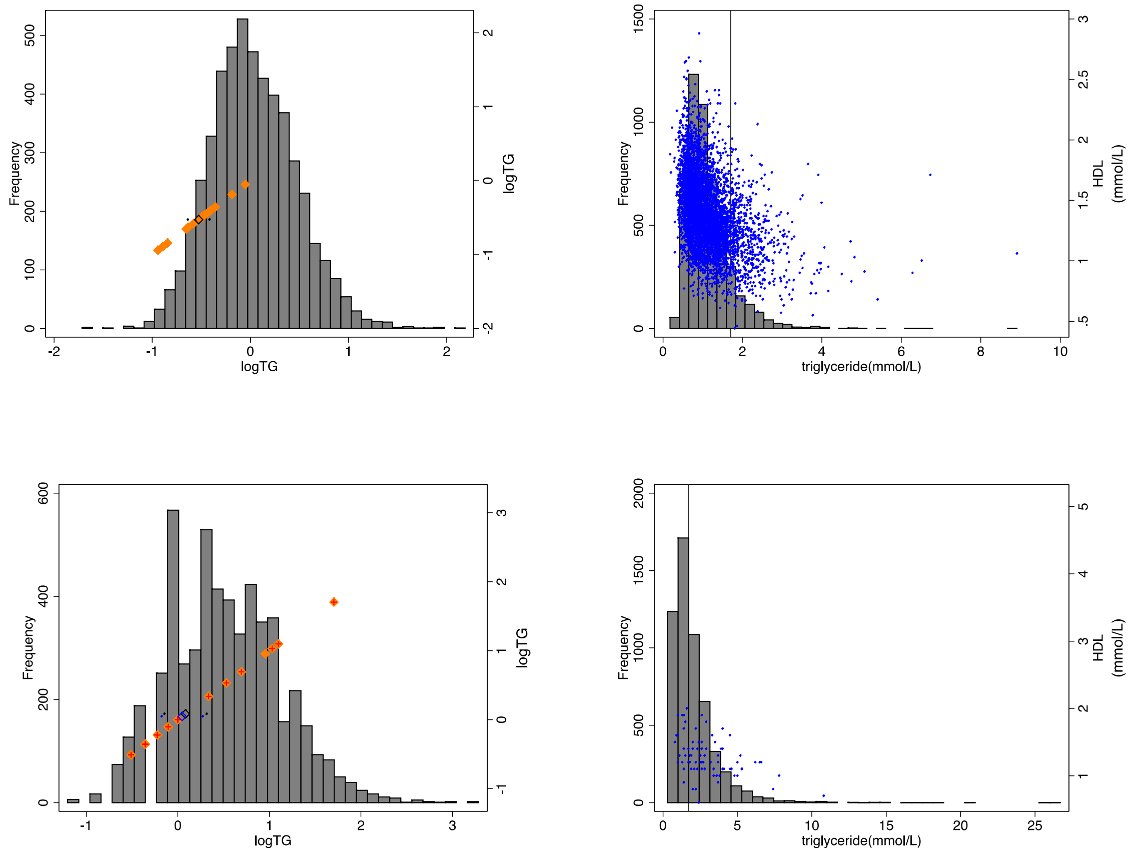

(a) Histogram of natural log transformed circulating triglyceride levels in ALSPAC participants (aged 9 years). Orange markers highlight the TG values of carriers of rs138326449 minor alleles (all heterozygotes, n=17 out of 5,040 with lipid data). Black diamond marks the mean natural log transformed triglyceride value for carriers with filled diamonds showing 95%CI for this estimate. (b) Vertical line indicates the current accepted (adult) threshold for hypertriglyceridaemia (<150 mg/dL (<1.7 mmol/L)) over-layed with the native distribution of triglycerides in ALSPAC participants and a scatter of their circulating HDL levels. Plots (c) and (d) are the same as (a) and (b), but derived from adults within the 1958 birth cohort (biomedical clinic run at age 42 years). (c) contains the additional stratification of rs138326449 minor allele carrier triglyceride status in the presence and absence of lipid lowering drugs (those adhering to treatment are plotted as red crossed for values and blue markers for summary).

Supplementary Figure 7. Predicted impact of splice donor variant rs138326449 on mRNA maturation.

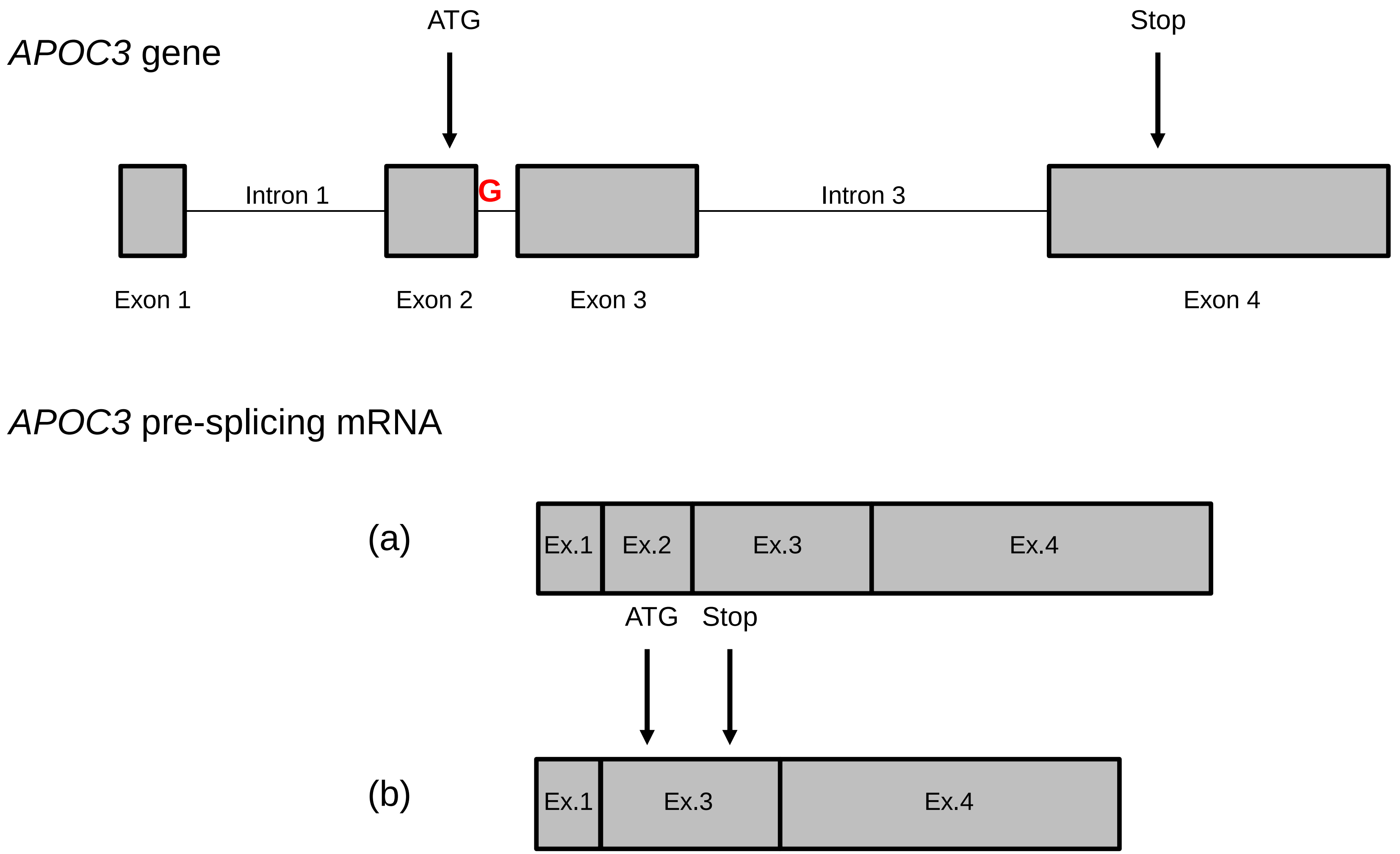

In the *APOC3* rs138326449 wild type (G marked in red) allele the gene is transcribed into mRNA composed of four exons (a). The first codon is located in the exon 2 of the gene whereas the stop codon is in the exon 4. Exon 1 and a part of exon 2 code for 5'UTR. In the *APOC3* rs138326449 minor (A marked in red) allele the exon 2 of the *APOC3* gene is likely to be skipped because its splicing donor site will no longer be recognised (b). The next ATG codon available is located in the exon 3. Translation from the ATG in the exon 3 will cause a frame shift and a formation of a premature stop codon after 22 amino acids with unlikely functionality.

Supplementary Tables

Supplementary Table 1. Characteristics of participating studies

Study	Country of Origin	Mean Age (yrs, range)	% Female	Genotype origin [1]	TG		VLDL		LDL		HDL		TC	
					N	Mean (SE)	N	Mean (SE)	N	Mean (SE)	N	Mean (SE)	N	Mean (SE)
**Discovery**														
ALSPAC WGS	UK	10 (9-11)	50	WGS	1497	1.14 (0.01)	1497	0.52 (0.01)	1495	2.31 (0.01)	1497	1.40 (0.01)	1495	4.24 (0.02)
TwinsUK WGS	UK	56 (17-85)	100	WGS	1705	1.12 (0.01)	1700	0.51 (0.01)	1696	3.16 (0.02)	1713	1.79 (0.01)	1711	5.48 (0.03)
**Replication**														
ALSPAC GWA	UK	10 (9-12)	49	IMP	2820	1.14 (0.01)	2820	0.52 (0.01)	2815	2.36 (0.01)	2820	1.40 (0.01)	2817	4.28 (0.01)
TwinsUK GWA	UK	50 (16-83)	81	IMP	1882	1.18 (0.02)	1874	0.53 (0.01)	1870	3.33 (0.03)	1896	1.51 (0.01)	1895	5.38 (0.03)
1958 Birth Cohort	UK	44 (44-44)	51-53	IMP	5485	2.07 (0.02)	-	-	5186	3.42 (0.01)	5493	1.56 (0.01)	5504	5.88 (0.01)
INCIPE	Italy	58 (26-95)	51	IMP	1382	1.10 (0.02)	-	-	1380	3.39 (0.02)	1382	1.49 (0.01)	1381	5.39 (0.03)
HELIC MANOLIS	Greece	62 (18-99)	57	IMP	1262	1.56 (0.03)	-	-	1,270	3.22 (0.03)	1264	1.32 (0.01)	1255	5.57 (0.08)

[1] WGS = whole genome sequence; IMP = imputed using the combined UK10K+1KGP reference panel

Supplementary Table 2. Details of trait transformation and statistical methods applied to each study

**Study**	**Transformation**	**Covariates** [1]	**Sample exclusion**	**Units**	**Fasting samples**	**Genotyping Platform(s)**	**Association testing software**
ALSPAC WGS	Inverse normal	Age	5SD	mmol/L	Non-fasting	Illumina HumanHap550	SNPtest[1]
TwinsUK WGS	Inverse normal	Age and age^2, analyser (random effect)	5SD	mmol/L	Fasting and non-fasting (96% fasting)	Illumina HumanHap300+Illumina Human610	SNPtest[1]
ALSPAC GWAS	Inverse normal	Age	5SD	mmol/L	Non-fasting	Illumina HumanHap550	SNPtest[1]
TwinsUK GWAS	Inverse normal	Age and analyser (random effect)	4SD	mmol/L	Fasting and non-fasting (87% fasting)	Illumina HumanHap300+Illumina Human610	GEMMA[2]
1958 Birth Cohort	Inverse normal	Age, age^2, sex and lipid lowering drugs	5SD	mmol/L	Non-fasting	Affymetrix 500K, Affymetrix v6.0, Illumina 1.2M chips and Illumina 660K, CardioMetabochip, Illumina 15k and Illumina HumanHap 550	SNPtest[1]
INCIPE	Inverse normal	Age, age^2	5SD +lipid lowering medication	mmol/L	NA	HumanCoreExome-12v1	SNPtest[1]
HELIC MANOLIS	Inverse normal	Age, age^2, fasting status	5SD	mmol/L	Fasting and non-fasting (66% fasting)	IlluminaOmniExpress700KBeadChip/ HumanExome-12v1	GEMMA[2]

[1] Covariates were applied to each strata where associated with lipid, as described in the trait transformation paragraph in the methods section.

Supplementary Table 3. Genetic associations between rs138326449 and total and HDL cholesterol levels.

**Test**	**Sample**	**TC**	**LDL**	**VLDL**
TwinsUK	EAF	0.0023	0.0023	0.0023
	Beta (SE)	-0.346 (0.396)	-0.391 (0.371)	-0.274 (0.103)
	P-value	0.382	0.292	7.71×10^{-3}
	N	1,711	1,696	1,700
	Info metric	0.78	0.78	0.78
ALSPAC	EAF	0.0028	0.0028	0.0028
	Beta (SE)	0.130 (0.228)	0.041 (0.206)	-0.236 (0.091)
	P-value	0.570	0.841	9.25×10^{-3}
	N	1,495	1,495	1,497
	Info metric	0.94	0.94	0.94
**Discovery combined**	**EAF**	**0.0025**	**0.0025**	**0.0025**
	**Beta (SE)**	**0.018 (0.263)**	**-0.063 (0.262)**	**-1.426 (0.265)**
	**P-value**	**0.944**	**0.811**	$\mathbf{7.82 \times 10^{-8}}$
	**N**	**3,205**	**3,191**	**3,197**
1958BC	EAF	0.0016	0.0016	-
	Beta (SE)	-0.015 (0.321)	0.012 (0.333)	-
	P-value	0.963	0.971	-
	N	5,504	5,186	-
	Info metric	0.55	0.55	-
INCIPE2	EAF	0.0026	0.0026	-
	Beta (SE)	0.190 (0.421)	0.249 (0.415)	-
	P-value	0.652	0.549	-
	N	1,381	1,380	-
	Info metric	0.78	0.78	-
TwinsUK	EAF	0.0029	0.0029	0.0029
	Beta (SE)	0.110 (0.339)	-0.052 (0.344)	-0.897 (0.357)
	P-value	0.746	0.879	0.012
	N	1,895	1,870	1874
	Info metric	0.75	0.75	0.75
ALSPAC	EAF	0.0010	0.0010	0.0010
	Beta (SE)	0.526 (0.569)	0.385 (0.570)	-1.828 (0.562)
	P-value	0.356	0.500	1.15×10^{-3}
	N	2,817	2,815	2,820
	Info metric	0.77	0.77	0.77
HELIC MANOLIS	EAF	0.0078	0.0078	-
	Beta (SE)	-0.392 (0.360)	0.703 (0.356)	-
	P-value	0.276	0.049	-
	N	1,255	1,270	-
	Info metric	0.42	0.42	-
**Replication combined**	**EAF**	**0.0013**	**0.0013**	**0.0018**
	**Beta (SE)**	**0.378 (0.169)**	**-0.095 (0.171)**	**-1.164 (0.301)**
	**P-value**	**0.026**	**0.579**	$\mathbf{1.12 \times 10^{-4}}$
	**N**	**12,850**	**12,519**	**4,694**
**Overall**	**EAF**	**0.0019**	**0.0019**	**0.0021**
	**Beta (SE)**	**0.273 (0.142)**	**-0.085 (0.143)**	**-1.312 (0.199)**
	**P-value**	**0.056**	**0.550**	$\mathbf{4.16 \times 10^{-11}}$
	**N**	**16,055**	**15,710**	**7,891**

EAF indicates the frequency of the effect allele. Beta (SE) are expressed in standard deviations units.

Supplementary Table 4. Independent contributing signals to singlepoint analyses within the *APOC3* region.

rsID	Chr:position	Reference allele	Effect allele frequency	Beta (SE)	P-value
rs193204541	11: 116,550,916	G	0.017	-0.37 (0.10)	$8.5x10^{-5}$
¥rs964184	11:116,648,917	G	0.133	0.21 (0.04)	$6.4x10^{-9}$
¥rs2075290	11:116,653,296	C	0.068	0.23 (0.05)	$1.8x10^{-6}$
				**Beta (SE) conditional**	**P-value conditional**
†rs138326449	11:116,701,354	A	0.002	-1.45 (0.27)	$4.8x10^{-8}$

Summary statistics are given for three variants independently associated with TG levels (based on GCTA conditional analysis).

†Analysis of splice donor variant rs138326449 conditional on genotype status at the three SNPs suggest an independent contribution to TG in this region.

¥ Indicates loci previously reported in association with plasma lipid-subfractions in genomewide association analysis[3,4].

Supplementary Table 5. Gene based SKAT analyses for the *APOC3* region.

Chromsomal window	Gene	Number of variants	ALSPAC_p	TwinsUK_p
115820932-115821474	*AP000797.1*	10	0.73	0.91
115821893-115822143	*AP000797.2*	10	0.99	0.78
116619032-116643694	*BUD13*	29	0.49	0.57
116648670-116658747	*ZNF259*	40	0.63	0.08
116660110-116663128	*APOA5*	21	0.15	0.25
116691446-116694005	*APOA4*	28	0.01	0.11
116700487-116703739	*APOC3*	9	0.01	0.03
116706477-116708253	*APOA1*	25	0.63	0.56
116714152-116719979	*SIK3*	31	0.01	0.41
116716003-116735115	*SIK3*	51	0.20	0.19
116727637-116735728	*SIK3*	43	0.17	0.33
116735256-116827748	*SIK3*	50	0.17	0.53
116738435-116827748	*SIK3*	46	0.16	0.50
116988551-116989853	*AP000936.5*	6	0.68	0.63
117015010-117040091	*PAFAH1B2*	41	0.05	0.36
117038657-117046781	*PAFAH1B2*	51	0.08	0.40
117040114-117047493	*PAFAH1B2*	37	0.24	0.41
117049476-117057351	*SIDT2*	38	0.49	0.55
117053229-117060458	*SIDT2*	51	0.69	0.78
117058341-117060955	*SIDT2*	42	0.23	0.95
117060470-117067185	*SIDT2*	50	0.83	0.32
117061407-117068139	*SIDT2*	62	0.92	0.20
117070088-117074090	*TAGLN*	41	0.44	0.31
117072339-117075425	*TAGLN*	42	0.79	0.58
117074525-117075425	*TAGLN*	14	0.95	0.47
117075158-117084038	*PCSK7*	58	0.45	0.61
117081441-117084963	*PCSK7*	51	0.07	0.57
117084060-117088435	*PCSK7*	37	0.19	0.26
117084974-117090146	*PCSK7*	50	0.17	0.70
117088494-117090832	*PCSK7*	35	0.09	0.96
117090246-117094845	*PCSK7*	50	0.94	0.04
117091044-117094846	*PCSK7*	44	0.94	0.04
117094846-117100750	*PCSK7*	50	0.93	0.95
117095456-117103172	*PCSK7*	58	0.95	0.97

Table S5 reports triglyceride specific SKAT results for genes contained within the APOC3 region along with counts of the contributing variants contained within them.

Supplementary Table 6. Summary of variance explained in circulating triglycerides from data of differing population samples.

Data set	N	Variance explained (%)
ALSPAC all	5,037	0.54%
ALSPAC WGS	1,541	0.82%
ALSPAC GWAS	3,502	0.39%
1958 Birth Cohort	5,486	0.27%

Estimates of TG variance explained for rs138326449 were derived from the ALSPAC and 1958 Birth Cohort collections using linear regression taking into account covariables age, age^2, sex and lipid lowering drugs in the case of the 1958 birth cohort.

Supplementary Table 7. Summary of association results for known single point positive controls.

SNP	Chr	N (reported)	Major allele	Minor allele	p (reported)	Source	p (UK10K)
rs12748152	1	178000	C	T	1.00E-09	GLC	1.49E-02
rs2131925	1	96598	T	G	9.00E-43	Teslovich	1.22E-03
rs1321257	1	92418	A	G	2.10E-14	Teslovich	6.54E-03
rs1042034	2	96590	T	C	1.00E-45	Teslovich	0.13
rs1260326	2	96590	C	T	6.00E-133	Teslovich	2.30E-05
rs10195252	2	96590	T	C	1.60E-10	Teslovich	1.58E-02
rs2943645	2	93554	T	C	2.40E-08	Teslovich	0.601066
rs645040	3	96597	T	G	2.50E-08	Teslovich	0.940662
rs6831256	4	177000	A	G	2.00E-12	GLC	0.998634
rs442177	4	96598	T	G	8.60E-12	Teslovich	4.06E-02
rs9686661	5	95848	C	T	1.30E-10	Teslovich	0.879865
rs2247056	6	96598	C	T	1.60E-15	Teslovich	0.152123
rs998584	6	175000	C	A	3.00E-15	GLC	0.154462
rs1936800	6	168000	T	C	3.00E-08	GLC	2.61E-02
rs4722551	7	178000	T	C	9.00E-11	GLC	2.76E-02
rs13238203	7	78797	C	T	1.10E-09	Teslovich	0.647128
rs7811265	7	96598	A	G	9.00E-59	Teslovich	8.58E-02
rs38855	7	178000	A	G	2.00E-08	GLC	0.126086
rs11776767	8	96598	G	C	1.30E-08	Teslovich	5.06E-02
rs1495743	8	96580	C	G	4.10E-14	Teslovich	0.303704
rs12678919	8	96598	A	G	2.00E-115	Teslovich	1.50E-05
rs2954029	8	96598	A	T	3.00E-55	Teslovich	4.60E-03
rs1832007	10	178000	A	G	2.00E-12	GLC	0.719685
rs10761731	10	96598	A	T	3.50E-12	Teslovich	0.385034
rs2068888	10	96598	G	A	2.40E-08	Teslovich	2.16E-02
rs174546	11	96598	C	T	5.40E-24	Teslovich	3.75E-02
rs964184	11	96576	C	G	7.00E-240	Teslovich	6.80E-09
rs11613352	12	96598	C	T	4.40E-10	Teslovich	0.539274
rs12310367	12	96598	A	G	1.20E-08	Teslovich	0.515349
rs2412710	15	86707	G	A	1.90E-08	Teslovich	0.793568
rs2929282	15	95070	A	T	1.60E-11	Teslovich	0.128233
rs261342	15	95070	C	G	2.40E-13	Teslovich	2.87E-02
rs3198697	16	176000	C	T	2.00E-08	GLC	0.698687
rs11649653	16	95034	C	G	3.40E-08	Teslovich	0.61931
rs1121980	16	155000	G	A	3.00E-08	GLC	0.133165
rs7205804	16	95070	G	A	1.20E-12	Teslovich	2.52E-02
rs8077889	17	176000	A	C	1.00E-08	GLC	0.60479
rs7248104	19	176000	G	A	5.00E-10	GLC	3.23E-02
rs10401969	19	95054	T	C	1.60E-29	Teslovich	8.85E-02
rs731839	19	176000	A	G	3.00E-09	GLC	0.309383
rs439401	19	65871	C	T	1.10E-30	Teslovich	1.40E-06
rs4810479	20	95070	T	C	4.70E-18	Teslovich	1.72E-02
rs5756931	22	95067	T	C	3.80E-08	Teslovich	0.192602

Teslovich secondary signals following conditional analysis on main genomewide signal.							
rs4660808	1	90819	T	C	3.00E-08	Teslovich secondary	0.118312
rs668948	2	91483	A	G	4.30E-10	Teslovich secondary	0.970446
rs636202	6	93855	T	C	2.60E-08	Teslovich secondary	0.127256
rs486359	6	93855	C	G	3.60E-09	Teslovich secondary	0.593714
rs1562398	7	93855	C	G	2.40E-08	Teslovich secondary	0.413473
rs7016529	8	81126	T	C	1.70E-29	Teslovich secondary	5.80E-02
rs261334	15	93855	C	G	1.60E-13	Teslovich secondary	2.61E-02
rs4803770	19	74315	C	G	2.90E-12	Teslovich secondary	0.191229

Table S6 reports triglyceride specific association results from both Teslovich et al and the Global Lipids Consortium as a reference to known singlepoint positive controls[3,4]. These are shown alongside p-values summarizing the evidence for association within the UK10K whole genome sequence discovery meta-analysis. Teslovich and GLC (Global Lipids Consortium) indicate the source of positive control result and where indicated results are from secondary variants surviving conditional analysis for either previously known signals or top results at that locus.

Supplementary Methods

Low read-depth whole genome sequencing (cohorts dataset)

Low read-depth whole-genome sequencing (WGS) was performed at both the Wellcome Trust Sanger Institute (WTSI) and the Beijing Genomics Institute (BGI). DNA (1-3μg) was sheared to 100–1000 bp using a Covaris E210 or LE220 (Covaris, Woburn, MA, USA). Sheared DNA was subjected to Illumina paired-end DNA library preparation. Following size selection (300-500 bp insert size), DNA libraries were sequenced using the Illumina HiSeq platform as paired-end 100 base reads according to manufacturer's protocol.

Alignment and BAM processing

Data generated at the Sanger Institute and BGI were aligned to the human reference separately by the respective centres. The BAM files[1] produced from these alignments were submitted to the European Genome-phenome Archive (EGA). The Vertebrate Resequencing Group at the Sanger Institute then performed further processing.

Alignment

Sequencing reads that failed QC were removed using the Illumina GA Pipeline, and the rest were aligned to the GRCh37 human reference, specifically the reference used in Phase 1 of the 1000 Genomes Project (ftp.1000genomes.ebi.ac.uk/vol1/ftp/technical/reference/human_g1k_v37.fasta.gz). Reads were aligned using BWA (v0.5.9-r16)[2]. This involved the following steps:

1. Index the reference fasta file:

bwa index -a bwtsw <reference_fasta>

2. For each fastq file:

bwa aln -q 15 -f <sai_file> <reference_fasta> <fastq_file>

3. Create SAM files [sam] using bwa sampe for paired-end reads:

bwa sampe -f <sam_file> <reference_fasta> <sai_files> <fastq_files>

4. Create sorted BAM from SAM. For alignments created at the Sanger this was done using Picard (v1.36) (http://picard.sourceforge.net/) SamFormatConverter and samtools (v0.1.11) sort. For alignments created at the BGI, this was done using samtools (v0.1.8) view and samtools sort.

5. PCR duplicates reads in the Sanger alignments were marked as duplicate using the Picard MarkDuplicates, while in the BGI alignments they were removed using samtools rmdup.

BAM improvement and sample file production

Further processing to improve SNV and INDEL calling, including realignment around known INDELs, base quality score recalibration, addition of BAQ tags, merging and duplicate marking follows that used for Illumina low-coverage data in Phase 1 of the 1000 Genomes Project[3]. Software versions used for UK10K for the steps described in that section were GATK version 1.1-5-g6f43284, Picard version 1.64 and samtools version 0.1.16.

Variant calling

SNV and INDEL calls were made using samtools/bcftools (version 0.1.18-r579)[4] by pooling the alignments from 3,910 individual low read-depth BAM files. All-samples and all-sites genotype likelihood files (bcf) were created with the samtools mpileup command:

samtools mpileup -EDVSp -C50 -m3 -F0.2 -d 8000 -P ILLUMINA -g –f <reference_fasta>

with the flags:

C=Coefficient for downgrading mapping quality for reads containing excessive mismatches.
d=At a position, read maximally *d* reads per input BAM

Variants were then called using the following bcftools command to produce a VCF file[5]

bcftools view -m 0.9 -vcgN.

For calling on chromosome X and Y, the following settings were applied. The pseudo-autosomal region (PAR) was masked on chromosome Y in the reference fasta file. Male samples were called as diploid in the PAR on chromosome X, and haploid otherwise. Diploid/haploid calls were made using the -s option in bcftools view. The PAR regions were: X-PAR1 (60,001-2,699,520); X-PAR2 (154,931,044-155,260,560); Y-PAR1 (10,001-2,649,520); Y-PAR2 (59,034,050-59,363,566).

The pipeline (run-mpileup) used to create the calls is available from https://github.com/VertebrateResequencing/vr-codebase/tree/develop.

Filtering

INDEL pre-filtering

The observation of spikes in the insertion/deletion ratio in sequencing cycles of a subset of the sequencing runs were linked to the appearance of bubbles in the flow cell during sequencing. To counteract this, the following post-calling filtering was applied. The bamcheck utility from the samtools package was used to create a distribution of INDELs per sequencing cycle. Lanes with INDELs predominantly clustered at certain read cycles were marked as problematic, specifically where the highest peak was 5x bigger than the median of the distribution. The list of problematic lanes included 159 samples. In the next step we checked mapped positions of the affected reads to see if they overlapped with called INDELs, which they did for 1,694,630 called sites. The genotypes and genotype likelihoods of affected samples were then set to the reference genotype unless there was a support for the INDEL also in a different, unaffected lane from the same sample. In total, 140,163 genotypes were set back to reference and 135,647 sites were excluded by this procedure. Note that this step was carried out on raw, unfiltered calls prior to VQSR filtering.

Site filtering

Variant Quality Score Recalibration (VQSR)[6] was used to filter sites. For SNVs, the GATK (version 1.3-21) UnifiedGenotyper was used to recall the sites/alleles discovered by samtools in order to generate annotations to be used for recalibration. Recalibration for the INDELs used annotations derived from the built-in samtools annotations. The GATK VariantRecalibrator was then used to model the variants, followed by GATK ApplyRecalibration, which assigns VQSLOD (variant quality score

log odds ratio) values to the variants. SNVs and INDELs were modeled separately, with parameters given below:

	**SNVs**	**INDELs**
Annotations	QD, DP, FS, MQ, HaplotypeScore, MQRankSum, ReadPosRankSum, InbreedingCoeff	MSD, MDV, MSQ, ICF, DP, SB, VDB
Training set	HapMap 3.3: hapmap_3.3.b37.sites.vcf, Omni 2.5M chip: 1000G_omni2.5.b37.sites.vcf	Mills-Devine, 1000 Genomes Phase I
Truth set	HapMap 3.3: hapmap_3.3.b37.sites.vcf	Mills-Devine
Known set	dbSNP build 132: dbsnp_132.b37.vcf	Mills-Devine

The truth set includes sites defined as truly showing variation from the reference (GRCh37). VQSLOD scores are calibrated by how many of the truth sites are retained when sites with a VQSLOD score below a given threshold are filtered out. For SNV sites a truth sensitivity of 99.5%, which corresponded to a minimum VQSLOD score of -0.6804 was selected, i.e. for this threshold 99.5% of truth sites were retained. For INDEL sites a truth sensitivity of 97%, which corresponded to a minimum VQSLOD score of 0.5939 was chosen. Finally, we also introduced the filter $p<10^{-6}$ to remove sites that failed the Hardy-Weinberg equilibrium (HWE, 302,388 sites removed) and removed sites with evidence for differential frequency (logistic regression p-value > 1e-2) between samples sequenced at BGI and WTSI (277,563 sites removed).

The VQSLOD score and other annotations from GATK (BaseQRankSum, Dels, FS, HRun, HaplotypeScore, InbreedingCoeff, MQ0, MQRankSum, QD, ReadPosRankSum, culprit) were copied back to the original samtools calls, excluding annotations which already existed in or did no apply to the samtools VCFs (DP and MQ, AC, AN). Each VCF further contained the filters LowQual (a low quality variant according to GATK) and MinVQSLOD (Variant's VQSLOD score is less than the cutoff). All sites that did not fail these filters were marked as PASS and brought forward to the genotype refinement stage.

To investigate the presence of batch effects, we computed the pairwise identity by state (IBS) metrics for a joint dataset of 3621 individuals using an LD-pruned genotype set of 2,203,581 markers and performed a multidimensional scaling analysis (MDS) on 10 dimensions (PLINK,v1.07, options: --indep-pairwise , window size: 5000 SNPs, step: 1000 SNPs, R^2 : 0.2; --mds-plot 10). Given the presence of structure by genotyping batch, we ran a genomewide association analysis for the binary variable "sequencing center" ("BGI" / "SANGER") using a logistic regression model. 335,982 SNPs were associated with batch at an inclusive threshold of p-value <= 0.01 and formed a list that were subsequently filtered out from the genotype set, removing the batch effect due to sequencing centre.

Post-genotyping sample QC

Of the 4,030 samples (1,990 TwinsUK and 2,040 ALSPAC) that were submitted for sequencing, 3,910 samples (1,934 TwinsUK and 1,976 ALSPAC) were sequenced and went through the variant calling procedure. Low quality samples were identified before the genotype refinement by comparing the samples to their GWAS genotypes using approximately 20,000 sites on chromosome 20. Comparing the raw genotype calls to existing GWAS data, we removed a total of 112 samples (64 TwinsUK and 48 ALSPAC) because of one or more of the following causes: (i) high overall discordance to SNP array data (>3%) (55 TwinsUK and 36 ALSPAC), (ii) heterozygosity rate > 3SD from population mean (1 TwinsUK and 1 ALSPAC), suggesting contamination (iii) no SNP array data available for that sample (7 TwinsUK and 0 ALSPAC) and (iv) sample below 4x mean read-depth (1 TwinsUK and 11 ALSPAC). Overall, 3,798 samples (1,870 TwinsUK and 1,928 ALSPAC) were brought forward to the

genotype refinement step (the number used in association analysis will of course vary according to phenotypic data available).

Genotype Refinement

The missing and low confidence genotypes in the filtered VCFs were refined out through an imputation procedure with BEAGLE 4, rev909[7]. The program was run with default parameters. VCFs were split into chunks each containing a maximum of 3,000 sites plus 1,000 sites in buffer regions, that is, 500 on either side. Multiallelic sites were included in the imputation. It took 882 CPU weeks to complete. After imputation, chunks were recombined using the vcf-phased-join script from the vcftools [vcftools] package.

Post-refinement sample QC

Additional sample-level QC steps were carried out on refined genotypes, leading to the exclusion of additional 17 samples (16 TwinsUK and 1 ALSPAC) because of one or more of the following causes: (i) non-reference discordance (NRD) with GWAS SNV data > 5% (12 TwinsUK and 1 ALSPAC), (ii) multiple relations to other samples (13 TwinsUK and 1 ALSPAC), (iii) failed sex check (3 TwinsUK and 0 ALSPAC). To identify these samples we pruned the WGS data to a set of independent SNVs and calculated genome-wide average identity by state between each pair of samples across the two cohorts. Samples were removed if they had more than 25 relations with IBS>0.125 (a high number of relationships may indicate contamination). The resulting set of contaminated samples corresponded almost completely to the set of samples with NRD>5%. This left a final set of 3,781 samples (1,854 TwinsUK and 1,927 ALSPAC). These VCF files were submitted to the EGA.

To exclude the presence of participants of non-European ancestry in our dataset, we merged a pruned dataset to the 11 HapMap3 populations[8] and performed a principal components analysis (PCA) using EIGENSTRAT[9]. A total of 44 participants (12 TwinsUK and 32 ALSPAC) did not cluster to the European (CEU) cluster of samples and were removed from association analyses. We further sought to flag related individuals for exclusion in association tests. Although association methods that account for family relatedness are available both for common and rare variant tests[10], we opted to remove these individuals for ease of analysis for ease and speed of analysis. Overall, 69 samples (36 TwinsUK and 33 ALSPAC) were flagged because of relatedness greater than third degree relatedness (IBD>0.125). Finally 63 co-twin samples (42 dizygotic and 21 monozygotic) and three duplicate samples were removed from TwinsUK. The final sequence data set that was used for the association analyses comprises 3,621 samples (1,754 TwinsUK and 1,867 ALSPAC).

Re-phasing

SHAPEIT2[11] was then used to rephase the genotype data. The VCF files were converted to binary ped format. Multiallelic and MAF<0.02% (singleton and monomorphic) sites were removed. Files were then split into 3Mbp chunks with +/-250kbp flanking regions. SHAPEIT (v2.r727) was used to rephase the haplotypes with the following command line option in phase mode:

> *--thread 4 --window 0.5 --states 200 --effective-size 11418 -B chr20.$chunk --input-map genetic_map_chr20_combined_b37.txt --output-log $log --output-max chr20.$chunk.hap.gz chr20.$chunk.sample*

vcf-gensample [vcftools] was used to combine the original VCF with new phase information. Sites not rephased with SHAPEIT had any existing phase information removed. vcf-phased-join was used to stitch the chunked VCFs back together with phase determined by matching overlapping heterozygous sites.

These are the final VCF files released for the project. An imputation reference panel in the IMPUTE2 format created from these VCF files are also made available.

Imputation from the combined UK10K + 1000 Genomes Panel

GW SNP array data

For each of the cohorts, we had additional GWA data available. For ALSPAC, 6,557 samples were measured on Illumina HumanHap550 arrays and passed QC (population stratification, sex check, heterozygosity and relatedness (IBS>0.125)). For TwinsUK, 2,575 samples were genotyped on Illumina HumanHap300 or Illumina Human610 arrays. These samples passed QC on relatedness (IBS>0.125), population stratification, heterozygosity, zygosity and sex checks. Samples from the imputed datasets were unrelated to the sequence datasets (IBS>0.125). Variants discovered through WGS of the TwinsUK and ALSPAC cohorts along with those known from 1000 Genomes were imputed into the full genome-wide association study genotyped cohorts increasing the sample size for single point association analysis to 12,724 subjects.

Imputation of UK10K & UK10K+1000GP data into SNP arrays

We imputed into available GWAS data using a combined UK10K as a reference and a UK10K+1000GP panel. We developed new functionality in IMPUTE2[12] that uses each reference panel to impute the missing variants in its counterpart, and then combine the two reference panels at the union set of sites. We tested the three reference panels for imputing three SNP array data, a sub-sample of 1,000 individuals from the UK10K WGS dataset, four European samples (3 CEU, 1 TSI) sequenced by Complete Genomics (CG, depth: 80X)[13], and an Italian isolate genotyped on core-exome SNP array. Using 3,781 UK10K genomes consistently improved imputation quality compared to using the 1000GP panel alone and whilst the combined UK10K+1000GP panel does not substantively increase imputation accuracy compared to UK10K panel alone, this does increase the total number of imputable variants[14].

Replication samples

ALSPAC GWAS. The ALSPAC GWAS dataset was imputed using the UK10K reference panel and drawn from the same cohort described earlier and includes all cohort participants that were not selected from whole-genome sequencing and for which genome-wide SNP arrays were available. Overall, 6,557 samples not contained in the WGS dataset were genotyped on Illumina HumanHap550 arrays and passed quality control metrics as described in[15]. Lipid measurements were as described earlier.

TwinsUK GWAS. The TwinsUK GWAS dataset was imputed using the UK10K reference panel and includes all participants from the TwinsUK with genome-wide SNP array data and not sharing family relatedness with the WGS dataset. A total of 2,575 study participants genotyped on Illumina HumanHap300 or Illumina Human610 arrays passed QC metrics and were available for analysis[16]. Lipid measurements were as described earlier. The majority of replication samples were fasted before measurement (87%).

1958 Birth Cohort. The 1958BC was imputed using the UK10K+1000GP reference panel. Participants to the cohort have been followed-up regularly since birth with prospective information collected on a wide range of indicators related to health, health behaviour, lifestyle, growth and development. There have been 9 contacts with the participants since their birth (ages 7, 11, 16, 23, 33, 41, 45, 47, and 50 years). The biomedical survey at age 45 years included collection of blood samples and DNA from about 8000 participants. The South East multicentre research ethics committee

(MREC) approved the study. There was an informed consent process conducted by the National Centre for Social Research [17].
Lipid measurements: Venous blood samples were obtained without prior fasting; participants could choose whether to sit or lie down when blood was taken. Serum TG, and total and HDL- cholesterol were measured in serum by Olympus model AU640 autoanalyser in a central lab in Newcastle. Enzymatic colorimetric determination GPO-PAP method was used to determine TGs, CHOD-PAP method for total cholesterol and for HDL-cholesterol. Blood samples were stored in a -80 C freezer.

INCIPE. INCIPE was imputed using the UK10K+1000GP reference panel. For the INCIPE study 6200 patients, all Caucasians, ≥40-years old by January 1, 2006, were randomly chosen from the lists of patients of 62 randomly selected general practitioners (GPs) based in four geographical areas in the Veneto region, NE Italy. In Italy all citizens receive free health insurance from the National Health System. To this aim all are included in the list of patients of GPs of their own choice. Thus, drawing participants from the GPs' lists is likely to draw them directly from the community. Enrolment and clinical examination were performed locally in four units, by trained medical doctors. A total of 3870 subjects (62%) accepted and were enrolled. Pregnant women were not enrolled. After written informed consent was obtained, each participant completed a self-administered questionnaire (e.g., family and personal medical history, pharmacologic treatments, and smoking habits). Patients were asked to refrain from smoking beginning from the night before. BP, waist circumference, body weight, and height and blood biochemistry were measured as in the original study [18]. The ethics committees of the involved institutions approved the study protocol.
Lipid measurements: Enzymatic determination of cholesterol and TGs was performed on the Dimension RxL apparatus (Siemens Diagnostics). HDL cholesterol was determined by the homogeneous method; LDL cholesterol was calculated using the Friedwald equation [19].

HELIC MANOLIS. The HELIC MENOLIS collection was imputed using the UK10K+1000GP reference panel. The HELIC (Hellenic Isolated Cohorts; www.helic.org) MANOLIS (Minoan Isolates) collection focuses on the Mylopotamos villages on Crete, Greece. Recruitment of this population-based sample was primarily carried out at the village medical centres. All individuals were older than 17 years and had to have at least one parent from the Mylopotamos area. The study includes biological sample collection for DNA extraction and lab-based blood measurements, and interview-based questionnaire filling. The phenotypes collected include anthropometric and biometric measurements, clinical evaluation data, biochemical and haematological profiles, self-reported medical history, demographic, socioeconomic and lifestyle information. The Harokopio University Bioethics Committee approved the study and informed consent was obtained from every participant.
Lipid measurements. Lipid traits were assessed using enzymatic colorimetric assays and included; total cholesterol (cholesterol oxidase - phenol aminophenazone method), high-density lipoprotein (HDL)-cholesterol and TGs (glycerol-3-phosphate oxidase -phenol aminophenazone). Low Density Lipoprotein (LDL)-cholesterol levels were calculated according to Friedewald equation [19]. Of the 1282 Helic Manolis individuals with lipid data, 849 were recorded as fasting when the biochemical measurements were taken.

Validation genotyping

For ALSPAC, the entire cohort (10,145 participants, including 38 carriers of the rare A allele) was genotyped using KASP at KBioscience (www.lgcgenomics.com/). Genotyping was undertaken at KBioscience where KASP genotyping was used. Assays are based on competitive allele-specific PCR and enable bi-allelic scoring of single nucleotide polymorphisms (SNPs) and insertions and deletions (Indels) at specific loci. The SNP-specific KASP Assay mix and the universal KASP Master mix are added to DNA samples, a thermal cycling reaction is then performed, followed by an end-point fluorescent read. Bi-allelic discrimination is achieved through the competitive binding of two allele-specific forward primers, each with a unique tail sequence that corresponds with two universal FRET

(fluorescence resonant energy transfer) cassettes; one labelled with FAMTM dye and the other with HEXTM dye.

For TwinsUK, genotyping accuracy was evaluated against a dataset comprising of ~250 high-coverage exomes sequenced in overlapping samples [20]. Of the 6 carriers detected in our study, four were overlapping and correctly called also in the exome dataset, yielding a genotyping accuracy of 100%.

There was 100% concordance with the genotypes called from the whole-genome dataset.

Supplementary References

1. Li, H. *et al.* The Sequence Alignment/Map format and SAMtools. *Bioinformatics* **25**, 2078-9 (2009).

2. Li, H. & Durbin, R. Fast and accurate long-read alignment with Burrows-Wheeler transform. *Bioinformatics* **26**, 589-95 (2010).

3. Consortium, T.G.P. An integrated map of genetic variation from 1,092 human genomes. *Nature* **491**, 56-65 (2012).

4. Li, H. A statistical framework for SNP calling, mutation discovery, association mapping and population genetical parameter estimation from sequencing data. *Bioinformatics* **27**, 2987-93 (2011).

5. Danecek, P. *et al.* The variant call format and VCFtools. *Bioinformatics* **27**, 2156-8 (2011).

6. DePristo, M.A. *et al.* A framework for variation discovery and genotyping using next-generation DNA sequencing data. *Nat Genet* **43**, 491-498 (2011).

7. Browning, S.R. & Browning, B.L. Rapid and accurate haplotype phasing and missing-data inference for whole-genome association studies by use of localized haplotype clustering. *American Journal of Human Genetics* **81**, 1084-97 (2007).

8. International HapMap, C. *et al.* Integrating common and rare genetic variation in diverse human populations. *Nature* **467**, 52-8 (2010).

9. Price, A.L. *et al.* Principal components analysis corrects for stratification in genome-wide association studies. *Nature Genetics* **38**, 904-9 (2006).

10. Zhou, X. & Stephens, M. Genome-wide efficient mixed-model analysis for association studies. *Nature Genetics* **44**, 821-824 (2012).

11. O'Connell, J. *et al.* A General Approach for Haplotype Phasing across the Full Spectrum of Relatedness. *PLoS Genet* **10**, e1004234 (2014).

12. Howie, B.N., Donnelly, P. & Marchini, J. A flexible and accurate genotype imputation method for the next generation of genome-wide association studies. *PLoS Genetics* **5**, e1000529 (2009).

13. Drmanac, R. *et al.* Human genome sequencing using unchained base reads on self-assembling DNA nanoarrays. *Science* **327**, 78-81 (2010).

14. Huang, J. *et al.* A reference panel of 3,781 genomes from the UK10K Project increases imputation performance of low frequency variants. *Nature Communications* **(under review)**(2014).

15. Paternoster, L. *et al.* Genetic Determinants of Trabecular and Cortical Volumetric Bone Mineral Densities and Bone Microstructure. *PLoS Genet* **9**, e1003247 (2013).

16. Suhre, K. *et al.* Human metabolic individuality in biomedical and pharmaceutical research. *Nature* **477**, 54-60 (2011).

17. Power, C. & Elliott, J. Cohort profile: 1958 British birth cohort (National Child Development Study). *Int J Epidemiol* **35**, 34-41 (2006).

18. Gambaro, G. *et al.* Prevalence of CKD in northeastern Italy: results of the INCIPE study and comparison with NHANES. *Clin J Am Soc Nephrol* **5**, 1946-53 (2010).

19. Friedewald, W.T., Levy, R.I. & Fredrickson, D.S. Estimation of the concentration of low-density lipoprotein cholesterol in plasma, without use of the preparative ultracentrifuge. *Clin Chem* **18**, 499-502 (1972).

20. Williams, F.M. *et al.* Genes contributing to pain sensitivity in the normal population: an exome sequencing study. *PLoS Genet* **8**, e1003095 (2012).
